# DNA Origami‐Cyanine Nanocomplex for Precision Imaging of KRAS‐Mutant Pancreatic Cancer Cells

**DOI:** 10.1002/advs.202410278

**Published:** 2025-02-14

**Authors:** Hye‐ran Moon, Yancheng Du, Sae Rome Choi, Seongmin Seo, Cih Cheng, Bennett D. Elzey, Jong Hyun Choi, Bumsoo Han

**Affiliations:** ^1^ School of Mechanical Engineering Purdue University West Lafayette IN 47907 USA; ^2^ Department of Mechanical Science and Engineering Department of Bioengineering University of Illinois Urbana‐Champaign Urbana IL 61801 USA; ^3^ Cancer Center at Illinois Materials Research Laboratory Institute of Genomic Biology Beckman Institute University of Illinois Urbana‐Champaign Urbana IL 61801 USA; ^4^ Purdue Institute for Cancer Research Purdue University West Lafayette IN 47907 USA; ^5^ Department of Comparative Pathobiology Purdue University West Lafayette IN 47907 USA; ^6^ Chan Zuckerberg Biohub Chicago Chicago IL 60642 USA; ^7^ Present address: Stem Cell Convergence Research Center Korea Research Institute of Bioscience and Biotechnology 125 Gwahak‐ro, Yuseong‐gu Daejeon 34141 Republic of Korea

**Keywords:** DNA origami, KRAS mutation, macropinocytosis, microfluidic tumor model, nanoparticle delivery, stroma tissue

## Abstract

Selective delivery of imaging agents to pancreatic cancer cells (PCCs) within the highly desmoplastic tumors of pancreatic ductal adenocarcinoma (PDAC) represents a significant advancement. This approach allows for precise labeling of PCCs while excluding cancer‐associated fibroblasts (CAFs), thereby enhancing both research and diagnostic capabilities. Additionally, it holds the potential to target and eliminate PCCs precisely without harming the surrounding stromal cells in the PDAC tumor microenvironment (TME). In this study, DNA origami‐cyanine (Do‐Cy) nanocomplexes are synthesized to image KRAS‐mutant PCCs selectively in the PDAC TME. These Do‐Cy nanocomplexes are hypothesized to be internalized preferentially to KRAS‐mutant PCCs over CAFs via elevated macropinocytosis. Several designs of Do‐Cy nanocomplexes are synthesized and characterized their cellular uptake using both engineered in vitro and xenograft pancreatic cancer models. The results are further discussed for the implication of precision delivery of therapeutic and imaging agents to KRAS‐mutant cancers.

## Graphical abstract

DNA origami‐cyanine (Do‐Cy) nanocomplexes are synthesized to selectively image KRAS‐mutant pancreatic cancer cells in the highly desmoplastic tumors. This study shows that the Do‐Cy nanocomplexes are preferentially internalized by cancer cells over other stroma cells via KRAS‐dependent macropinocytosis. The Do‐Cy nanocomplexes offer potential for molecular precision delivery of therapeutic and imaging agents to cancer cells with oncogenic KRAS mutations.

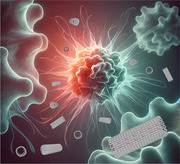

## 1 Introduction

Pancreatic ductal adenocarcinoma (PDAC) poses a significant clinical challenge with extremely low 5‐year survival rates of 13%.^[^ ^1^ ^]^ The PDAC tumor microenvironment (TME), characterized by a desmoplastic stroma that facilitates tumor growth, drives drug resistance and impedes drug delivery to pancreatic cancer cells (PCCs), causing poor treatment outcomes. The PDAC stroma accounts for up to 90% of the PDAC tumor volume^[^ ^2^, ^3^ ^]^ and is predominantly composed of cancer associated fibroblasts (CAFs) and extracellular matrix (ECM) components.^[^ ^4^, ^5^ ^]^ CAFs synthesize and remodel the stromal ECM, which closely contributes to tumorigenesis,^[^ ^6^, ^7^ ^]^ impedes drug delivery and correlates with poor prognosis.^[^ ^8^ ^]^ CAFs also regulate ECM degradation by expressing numerous proteases (e.g., secreted serine proteases and matrix metalloproteinases) that drive tumor growth and metastasis.^[^ ^9^, ^10^, ^11^ ^]^ Various growth factors secreted by CAFs also enhance an epithelial‐mesenchymal transition (EMT) and therapeutic resistance of PCCs.^[^ ^12^, ^13^ ^]^ The combination of these complex biochemical and biophysical mechanisms with heterogeneous genetic mutations makes PDAC extremely difficult to treat and diagnose. Although surgical resection of tumor is still the best treatment option for pancreatic cancer, PDAC tumors frequently exhibit poorly defined margins between malignant tissue and normal pancreas, contributing to a high risk of incomplete resection and local recurrence.^[^ ^14^, ^15^, ^16^ ^]^ Image‐guidance of surgical margin can be clinically useful, such as intraoperative fluorescence‐guided surgery.^[^ ^17^, ^18^ ^]^ Thus, the development of selective imaging agents and techniques for tumor cells could enable precise intraoperative visualization and removal of malignant PCCs while preserving surrounding normaltissues. This approach will reduce the risk of disease recurrence, maintain organ function, and lower postoperative complications.

To overcome the challenges in PDAC, targeted delivery of imaging and therapeutic agents has been extensively explored, leveraging PDAC‐specific antibodies or nanoparticle engineering to achieve tumor‐specific delivery to cancer cells. Various nanoparticle carriers have been utilized to load cytotoxic drugs and imaging agents for tumor‐selective delivery,^[^ ^19^ ^]^ including nucleic acid based,^[^ ^20^, ^21^, ^22^ ^]^ polymeric,^[^ ^23^, ^24^ ^]^ lipidic,^[^ ^25^, ^26^ ^]^ and inorganic vehicles.^[^ ^27^, ^28^ ^]^ Among various types of engineered nanomedicines, DNA nanocarriers have advanced rapidly with tremendous potential. DNA origami is a self‐assembly approach that can form arbitrary nanostructures from a long single‐stranded (ss) DNA (termed scaffold) with dozens of oligonucleotides (staples).^[^ ^29^ ^]^ Various origami constructs of different sizes and shapes have been demonstrated, including smiley faces, Mona Lisa, and polyhedral architectures, to name a few.^[^ ^29^, ^30^, ^31^, ^32^, ^33^ ^]^ They hold significant opportunities for imaging and drug delivery applications with notable features,^[^ ^21^, ^34^, ^35^, ^36^ ^]^ including the ability to address with various molecules,^[^ ^37^, ^38^, ^39^ ^]^ versatility in chemical modifications,^[^ ^40^, ^41^ ^]^ flexibility in structural and functional design,^[^ ^42^, ^43^ ^]^ and biocompatibility and stability.^[^ ^31^, ^44^ ^]^ Novel anti‐cancer DNA origami systems offer significant promise to enhanced targeting specificity, improved drug delivery efficiency, and reduced toxicity.^[^ ^21^, ^45^ ^]^ By precisely targeting cancer cells and minimizing exposure to healthy cells, DNA origami could become a highly effective approach to cancer treatment.^[^ ^35^, ^46^ ^]^

Despite significant advances, the translation of DNA‐origami nanocarriers into practical utility in targeted delivery has been largely hindered by the lack of sophisticated evaluation of tumor‐targeting delivery mechanisms. Specifically, understanding the tumor‐targeting specificity at the tissue level poses a significant challenge, as highly heterogeneous PDAC includes a substantial population of CAFs. Recent studies evaluated transport features of DNA‐origami carriers with inconsistent observations. For example, Wang et al. studied cellular uptake and trafficking of DNA origami with gold nanoparticle tags, examining various sizes, shapes, and surface chemistry.^[^ ^46^ ^]^ They found that larger origami nanostructures generally demonstrated greater uptake. While scavenger receptors were important in mediating cell uptake, no specific endocytosis pathway was identified. Other reports suggested that more compact structures promote cellular uptake efficiency.^[^ ^47^, ^48^, ^49^ ^]^ Another study constructed various DNA nanostructures (tetrahedron, cube, icosahedron, and buckyball) and observed a clathrin‐mediated pathway for cellular entry.^[^ ^50^ ^]^ Additionally, other studies have provided evidence of DNA nanostructures being transported into cells through alternative mechanisms, including caveolae‐mediated endocytosis in HeLa cells.^[^ ^46^ ^]^ Overall, uptake mechanisms are highly dependent on the cell lines and there is a lack of adequate information on DNA origami's targeting specificity in the complex and heterogeneous PDAC microenvironment nature. In‐depth studies on targeting efficacy and specificity of DNA origami‐based delivery are thus critically needed.

In this study, we developed DNA origami‐Cyanine (Do‐Cy) nanocomplexes by conjugating Cy5 and Cy3 dyes to selectively image KRAS‐mutant PCCs in the desmoplastic PDAC TME. This Do‐Cy nanocomplex is designed based on a rationale that KRAS‐mutant PCCs show elevated macropinocytosis than other stromal cells in the TME. Macropinocytosis is a non‐selective endocytic uptake of adjacent materials and is hyperactivated in KRAS‐mutant cancer cells to meet their increased metabolic demands in nutrient‐scarce environments.^[^ ^51^ ^]^ KRAS mutations, present in up to 95% of PDAC cases, are well‐established drivers of macropinocytosis. This study hypothesizes that the elevated macropinocytic activity in KRAS‐mutant pancreatic cancer cells facilitates the preferential internalization of DNA origami nanostructures, enabling selective targeting, specifically over stroma cells including CAFs. To test this hypothesis, we investigate the tumor‐selective uptake of DNA origami in the PDAC TME by using both in vitro and in vivo PDAC tumor models developed for mimicking the PDAC stromal microenvironment. The hypothesis is tested to establish a mechanistic comprehensive of the transport mechanisms of DNA origami having tumor specificity within this complex PDAC TME, characterized by a high degree of heterogeneity and the presence of large populations of CAFs. In addition to macropinocytosis, DNA nanostructures are reported to enter cells through diverse mechanisms, specifically clathrin‐mediated endocytosis (CME).^[^ ^50^ ^]^ CME is indirectly influenced by KRAS mutations through the activation of RAS‐RAF‐MEK‐ERK and PI3K‐Akt pathways,^[^ ^52^ ^]^ which enhance receptor tyrosine kinase (RTK) signaling and promote receptor recycling to support the metabolic demands of KRAS‐driven cancers.^[^ ^53^ ^]^ We further investigate the effects of both endocytosis mechanisms in KRAS‐mutant PDAC tumor models through pharmacological inhibition and comparative studies in KRAS‐mutant and wild‐type cell lines. The results are discussed for implication of precision delivery of therapeutic and imaging agents to KRAS‐mutant cancers.

## 2 Results

### 2.1 Synthesis and Characterization of DNA Origami‐Cyanine (Do‐Cy) Nanocomplex

**Figure 1:**
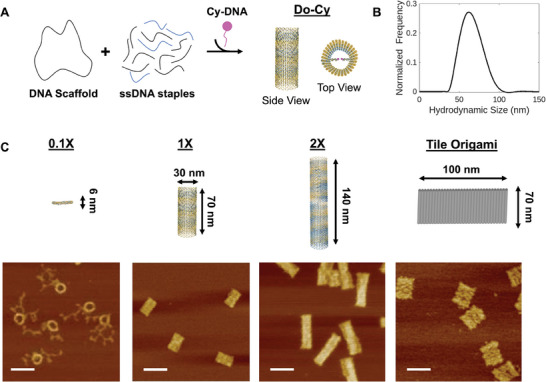
Tubular DNA origami nanostructures. A) Schematics of DNA origami labeled with Cy dyes (Do‐Cy). A tubular DNA origami is assembled from a long single‐stranded scaffold and dozens of oligo staples. Cy modified oligonucleotides are used for fluorescence imaging. B) DLS measurement shows an average hydrodynamic size of ≈63 nm. C) Schematics and AFM images of different sizes and shapes of DNA origami used. 1X Tubular DNA structures collapse onto mica surface for AFM imaging, measuring a thickness of ≈4 nm, length of ≈70 nm, and a width of ≈50 nm (corresponding to ≈30 nm diameter). Scale bar: 100 nm.

In this study, we design tube‐shaped DNA origami as a nanocarrier for cellular uptake (**Figure** **1**A). The template DNA scaffold and ss‐staples assembled into in‐silico‐designed structures via self‐assembly under thermal annealing. Thirty staples were modified with an extension for binding with oligos with Cyanine (Cy) fluorophores, thus ensuring 30 dyes per origami. DNA assemblies are purified with filtration to remove excessive ssDNA. The structural details and assembly conditions are discussed in the supplementary information and can also be found elsewhere.^[^ ^43^ ^]^ The designed 1X origami has a length of ≈70 nm and a diameter of ≈30 nm. The assembled DNA tubes are characterized with atomic force microscopy (AFM) and dynamic light scattering (DLS) as shown in Figure 1B,C. AFM measures a length of ≈70 nm, a width of ≈50 nm, and a thickness of ≈4 nm. These dimensions are expected given that the tile shape origami collapses onto mica surface, with the width corresponding to ≈30 nm diameter. Their measured hydrodynamic size is ≈63 nm, consistent with our design and AFM. Zeta potential was ≈‐15 mV (Figure S1, Supporting Information). We also constructed another two types of 30 nm‐diameter DNA tubes with variable lengths: twice long (≈140 nm; termed 2X) and about one tenth of the length (≈6 nm; termed 0.1X). Characterizations of these DNA nanostructures are included in the Supporting Information (See Tables S1–S5, Supporting Information). Three types of DNA tubules are used for cellular uptake in this study.

### 2.2 Characteristics of Cellular Uptake of the Do‐Cy by Pancreatic Cancer Cells and CAFs

**Figure 2:**
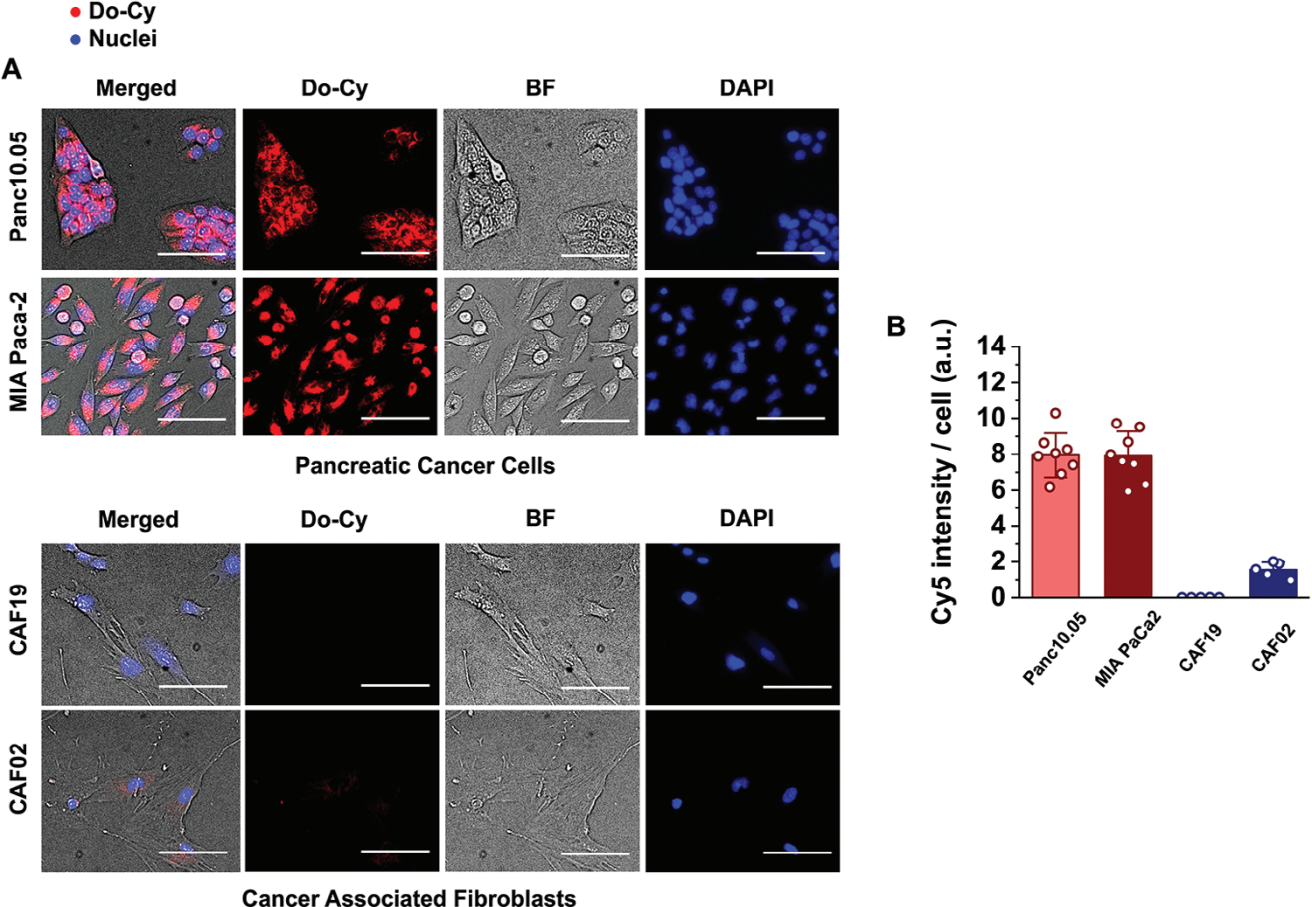
Do‐Cy nanocomplex accumulate in pancreatic tumor cells preferentially more than in stroma cells. A) Fluorescence micrograph of Do‐Cy accumulation after 24 h exposure in 2D monolayer of pancreatic cancer cells (Panc10.05 and MIA PaCa‐2) and CAFs (CAF19 and CAF02). B) Quantification of fluorescence demonstrates Do‐Cy accumulates significantly more in PCCs than CAFs. Bars indicate Mean ± S.E., and dots represent each data point (n ≥ 5). Scale bars indicate 100 µm.

We assess the cellular uptake characteristics of Do‐Cy nanocomplex in vitro using various PCCs and CAFs. The PCC panel includes Panc10.05, MIA PaCa‐2, and the CAF panel includes CAF19 and CAF02. The uptake of the Do‐Cy is captured with the fluorescence intensity of Cy accumulated on mono‐cultured cells after 24 h. **Figure** **2**A, B shows that the Do‐Cy nanocomplex accumulates in the cancer cells significantly more than in CAF cells.

**Figure 3:**
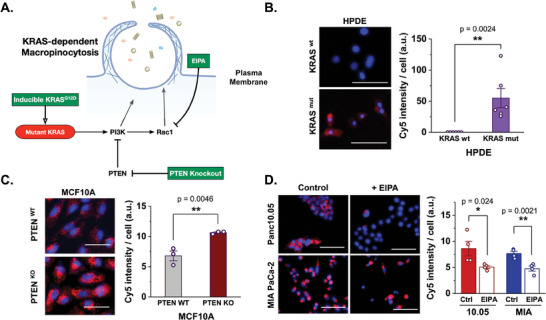
Do‐Cy accumulates in PCC through KRAS mutation dependent macropinocytosis. A) Experimental design. KRAS mutation in cancer cells result in the activation of PI3K and Rac1 pathway leading to elevated macropinocytosis. The experiments were designed to test these mechanisms – inducing KRAS mutation, genetic knockout of PTEN, a negative regulator of PI3K, and pharmacological inhibition of Rac1 using EIPA. B) Do‐Cy accumulation in KRAS mutation‐inducible human pancreatic duct epithelial cells (HPDE iKRAS): KRAS mutant cells (KRAS mut, treated with 25 ng mL^−1^ doxycycline for 48 h) and KRAS wild‐type cells (KRAS wt., treated with 1:100 dilution of DMSO in HPDE media). Red represents Do‐Cy, nuclei are blue, and GFP‐transfected CAFs are green. C) PTEN knockout in MCF10A, a KRAS mutant cell line, demonstrates elevated uptake of Do‐Cy indicating elevated KRAS mutation leads to heightened macropinocytosis. D) Do‐Cy accumulation in pancreatic cancer cells with and without EIPA, a macropinocytosis inhibitor. Bars indicate Mean ± S.E., and dots represent each data point (n ≥ 3). P‐values of <0.05, <0.01, and <0.001 are represented as *, **, and ***, respectively (Student t‐test). Scale bars indicate 100 µm.

To elucidate the mechanism of tumor‐preferential accumulation of DNA origami‐Cy, we hypothesize that macropinocytosis derived by oncogenic KRAS transformation in pancreatic cancer plays a key role in tumor‐selective uptake of DNA origami‐Cy. Elevation of KRAS mutation leads to activation of PI3K and Rac1 pathway leading to enhanced macropinocytosis (**Figure** **3**A). This pathway may also be inhibited by tumor suppressor gene, PTEN. The majority of pancreatic cancer patients possess oncogenic KRAS mutations that lead to the activation of macropinocytosis in cancer.^[^ ^51^, ^54^ ^]^ To test the effect of KRAS mutation on Do‐Cy uptake for cancer cells, we measure the accumulation of the conjugate on the genetically engineered cell lines, KRAS mutation‐inducible human pancreatic duct epithelial cells (HPDE iKRAS). HPDE iKRAS cells are developed to induce the KRAS mutation (KRAS G12D) in accordance with doxycycline treatment.^[^ ^55^ ^]^ KRAS mutant HPDE cells (KRAS mut) are induced with 25ng mL^−1^ doxycycline treatment whereas the control group with normal media is considered as KRAS wild type (KRAS wt.). Consequently, we observe that the Do‐Cy accumulates in KRAS mut cells significantly more than KRAS wt. cells (Figure 3B), which is consistent with our hypothesis that the elevated uptake of the DO‐Cy by PCC is caused by KRAS mutation‐dependent macropinocytosis. The hypothesis is further supported by MCF10A cell line whose Do‐Cy uptake is intensified upon PTEN knockout, which is RAS pathway suppressor (Figure 3C). This result suggests that increased RAS associated pathway activity may lead to elevated macropinocytosis of Do‐Dy.

Pharmacological inhibition of macropinocytosis of the cancer cells, Panc10.05 and MIA PaCa‐2, is achieved by inhibiting 5‐[N‐ethyl‐N‐isopropyl] amiloride (EIPA).^[^ ^56^ ^]^ As shown in Figure 3D, Do‐Cy accumulation in cancer cells is highly suppressed in EIPA‐treated groups in both cancer cell lines, supporting the role of macropinocytosis in the selective uptake of the Do‐Cy nanocomplex by cancer cells.

Further, Do‐Cy intensity demonstrates linear relationship with its prepared concentrations (Figure S2, Supporting Information), thereby suggesting measurement of Do‐Cy uptake based on their intensity is a reasonable method quantification. Established calibration curve could also be further utilized to estimate the intracellular concentration of accumulated Do‐Cy for future applications.

**Figure 4:**
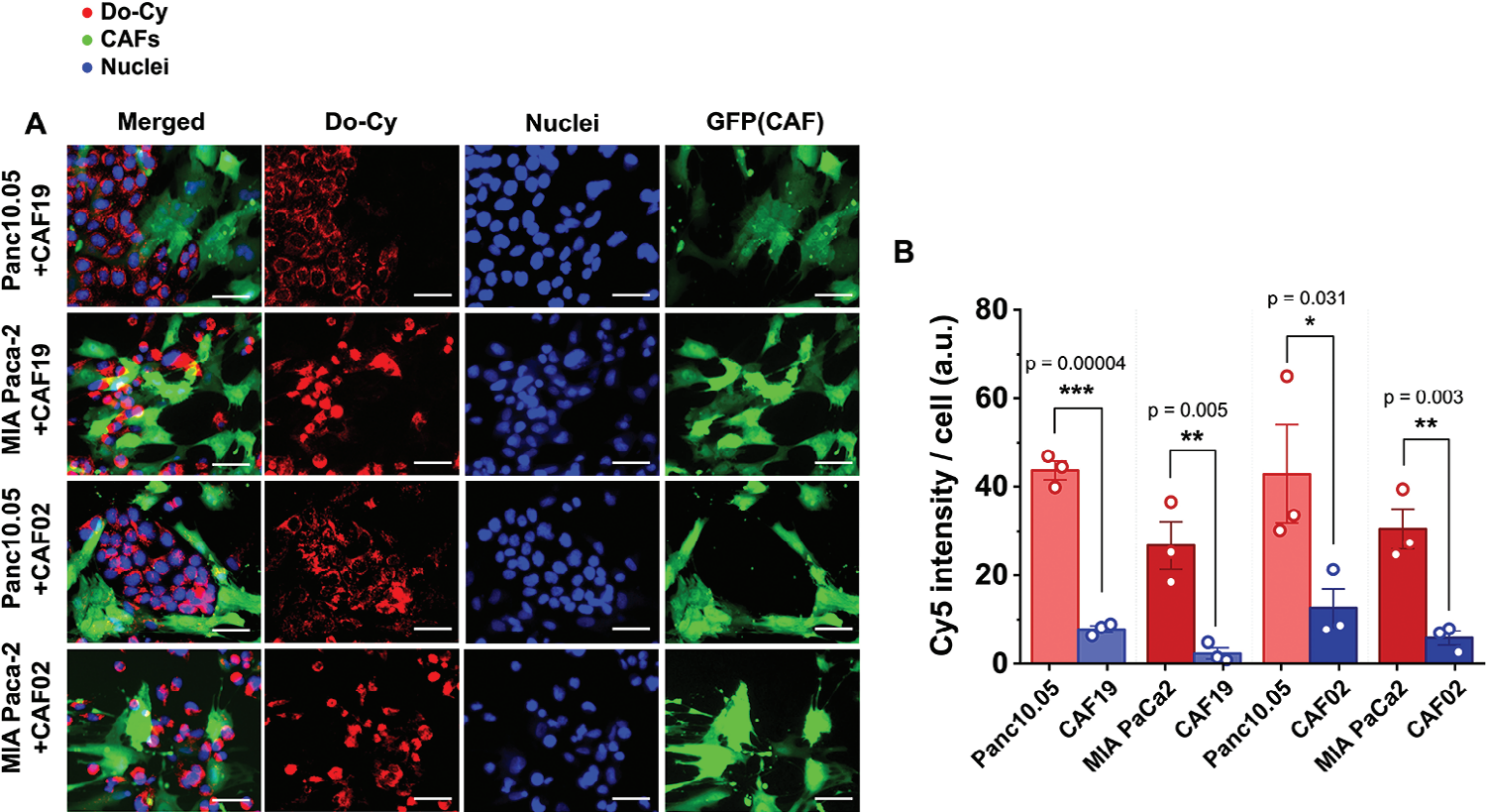
Do‐Cy nanocomplexes preferentially accumulate in pancreatic tumor cells over the CAF cells in co‐cultured PCC‐CAF. A) Fluorescence micrograph of Do‐Cy accumulation after 24 h exposure in co‐cultured monolayer pairing pancreatic cancer cells and CAFs (Panc10.05+CAF19, MIA PaCa‐2+CAF19, Panc10.05+CAF02, and MIA PaCa‐2+CAF02). B) Accumulated intensity of Cy phosphors conjugated on DNA origami normalized by the cell counts. Red represents Do‐Cy, nuclei are blue, and GFP‐transfected CAFs are green. Bars indicate Mean ± S.E., and dots represent each data point (n = 3). P‐values of <0.05, <0.01, and <0.001 are represented as *, **, and ***, respectively (Student t‐test). Scale bars indicate 100 µm.

Furthermore, we evaluate the tumor‐preferential accumulation of the Do‐Cy using the 2D co‐culture models of the PCCs and CAFs. Consistent with findings from the monoculture assays, we observe a significantly higher accumulation of Do‐Cy in the cancer cells compared to the CAF cells (**Figure** **4**). Specifically, Cy signals exhibit notably elevated expression in Panc10.05 cells within both Panc10.05+CAF19 and Panc10.05+CAF02 pairs. This trend is consistently observed across other cancer cell types, further supporting the tumor‐selective accumulation patterns of the Do‐Cy nanocomplex. We also confirm that the effect is not Cy phosphor‐specific by demonstrating that the DNA origami‐Cy nanocomplex exhibits similar tumor‐selective accumulation patterns (Figure S3, Supporting Information). The macropinocytosis‐induced tumor‐selective accumulation of Do‐Cy is additionally investigated in a co‐culture pair of Panc10.05 and CAF19. We observe that the intensity of Cy is significantly reduced in the EIPA treated group (Figure S4, Supporting Information). To confirm the reduction is due to the inhibition of macropinocytosis by EIPA but not due to the cell death by EIPA, we investigate the cell viability of Panc10.05 and CAF19, showing that there are no significant changes in viability after EIPA treatment (Figure S5, Supporting Information). The results indicate that the reduction of Do‐Cy accumulation is less associated with metabolic activities, supporting our hypothesis that macropinocytosis is a more likely contributor to the tumor‐selective uptake for Do‐Cy. While EIPA results show cancer cells primarily depend on macropinocytosis for Do‐Cy uptake, cancer cells may have other endocytosis mechanisms. The possibility of additional uptake mechanism, such as clathrin‐mediated endocytosis (CME), is also supported by the observation that the EPIA treatment does not completely block Do‐Cy uptake. However, the pharmacological inhibition of CME with a CME inhibitor, chlorpromazine, shows no significant decrease in the Do‐Cy uptake (Figure S6, Supporting Information). All these results suggest that macropinocytosis is a primary route of Do‐Cy nanomaterial preferential uptake for KRAS‐mutant PDAC cells. But further research is still warranted to identify other uptake mechanisms.

Furthermore, for applications of Do‐Cy in various precision imaging, it is critical to confirm their stability from various cellular components. To verify this, additional stability testing is performed by incubating Do‐Cy in PCC lysates.^[^ ^44^, ^57^ ^]^ Agarose gel electrophoresis shows a distinct band of DNA origami, and AFM images of the band clearly show DNA tubules (Figure S7, Supporting Information). The results suggest the excellent stability of Do‐Cy in PCCs and further prove the nanocomplex as a reliable material for application in imaging and delivery of other biological agents.

### 2.3 Effects of the Size and Shape of DNA Origami on the Cellular Uptake

DNA origami presents promising opportunities for engineering the size and shape of nanoparticles to enhance targeted delivery efficiency. Numerous studies have highlighted the crucial role of particle size in triggering endocytic processes, emphasizing the importance of optimizing size parameters for effective delivery systems.^[^ ^20^ ^]^ To evaluate the impact of Do‐Cy size, we hypothesize there exists an optimal size range that activates macropinocytosis. We thus vary the length of tubular Do‐Cy while keeping the diameter constant. The tubular origami tested are 1) ≈70 nm in length and 30 nm in diameter (1X size), 2) ≈140 nm in length and 30 nm in diameter (2X size), and 3) ≈6 nm in length and 30 nm in diameter (0.1X size). These nanostructures are exposed to the co‐cultured PCC and CAFs of Panc10.05‐CAF19 pairs and the accumulation patterns are transiently monitored.

**Figure 5:**
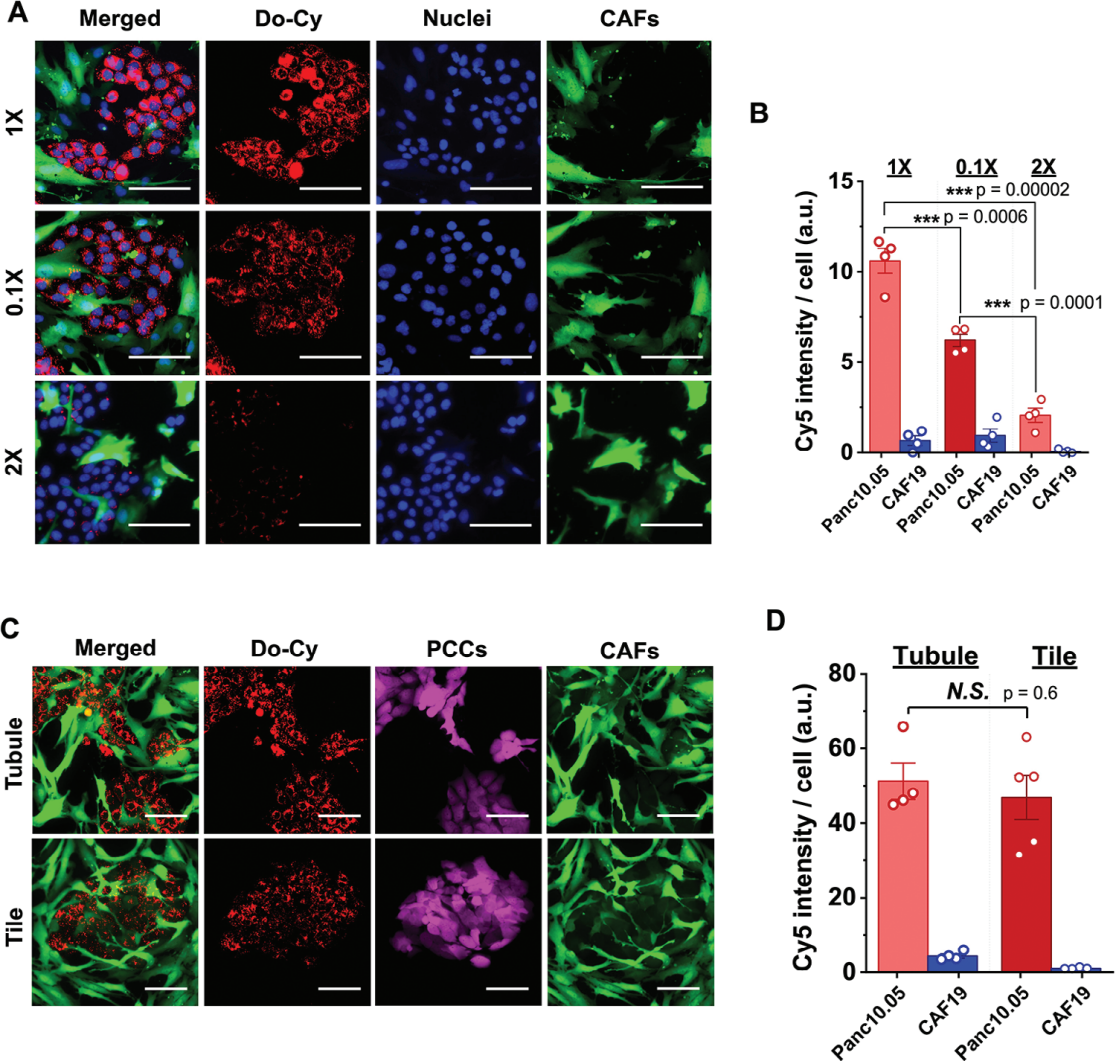
Effect of size and shape of the DNA origami on the tumor‐selective accumulation over CAFs. Fluorescent micrographs with A) three size variants of 1X, 0.1X, and 2X and B) quantification. Fluorescent micrographs with C) two shape variants of tubule and tile of DNA origami and D) quantification. Red represents accumulated Do‐Cy, green indicates transfected CAFs, blue indicates nuclei, and magenta indicates Td‐tomato labeled PCC (Panc10.05). Bars indicate Mean ± S.E., and dots represent each data point (n = 4). P‐values of <0.05, <0.01, and <0.001 are represented as *, **, and ***, respectively (Student t‐test). Scale bars indicate 100 µm.

The results demonstrate that all sizes of Do‐Cy (1X, 0.1X, and 2X) exhibit significantly higher accumulation in cancer cells compared to CAFs (**Figure** **5**A,B). Notably, we observe size‐dependency in the accumulation of Do‐Cy in pancreatic cancer cells. Do‐Cy at 1X and the smaller variant at 0.1X exhibit similar accumulation patterns. In contrast, the larger 2X variant shows significantly lower accumulation, indicating a size‐dependent variation in the cellular uptake efficiency of the cancer cells. This observation highlights the importance of optimizing DNA origami size to maximize the targeted delivery efficacy.

Furthermore, we explore the effect of altering the shape of DNA origamis on tumor‐specificity. Various geometries, such as tubules, tiles, and triangular DNA origami structures, have been developed as potential candidates for enhancing targeted delivery efficiency and specificity.^[^ ^34^, ^35^, ^58^ ^]^ We specifically focus on two representative shapes with similar sizes – tubules (1X) and tiles (100 nm × 70 nm), to compare potential differences in their transport patterns toward tumor‐specific accumulation in the tumor tissue. As presented in Figure 5C,D, the similar trends in the Do‐Cy delivery reveal that the shape of the origami is not a crucial contributor to delivery patterns. This further suggests that the tumor specificity of Do‐Cy delivery is more closely associated with the size of DNA origami structures rather than their shape, particularly in the context of selectivity to the cancer cells and CAFs. Further in vivo investigation would be required to comprehensively evaluate the tumor specificity compared to other organs.

### 2.4 Transport and Uptake Characteristics of Do‐Cy Nanocomplex in Engineered In Vitro and In Vivo PDAC Tumor‐Stroma Models

#### 2.4.1 Tumoroids with Tumor–Stroma Interface

**Figure 6:**
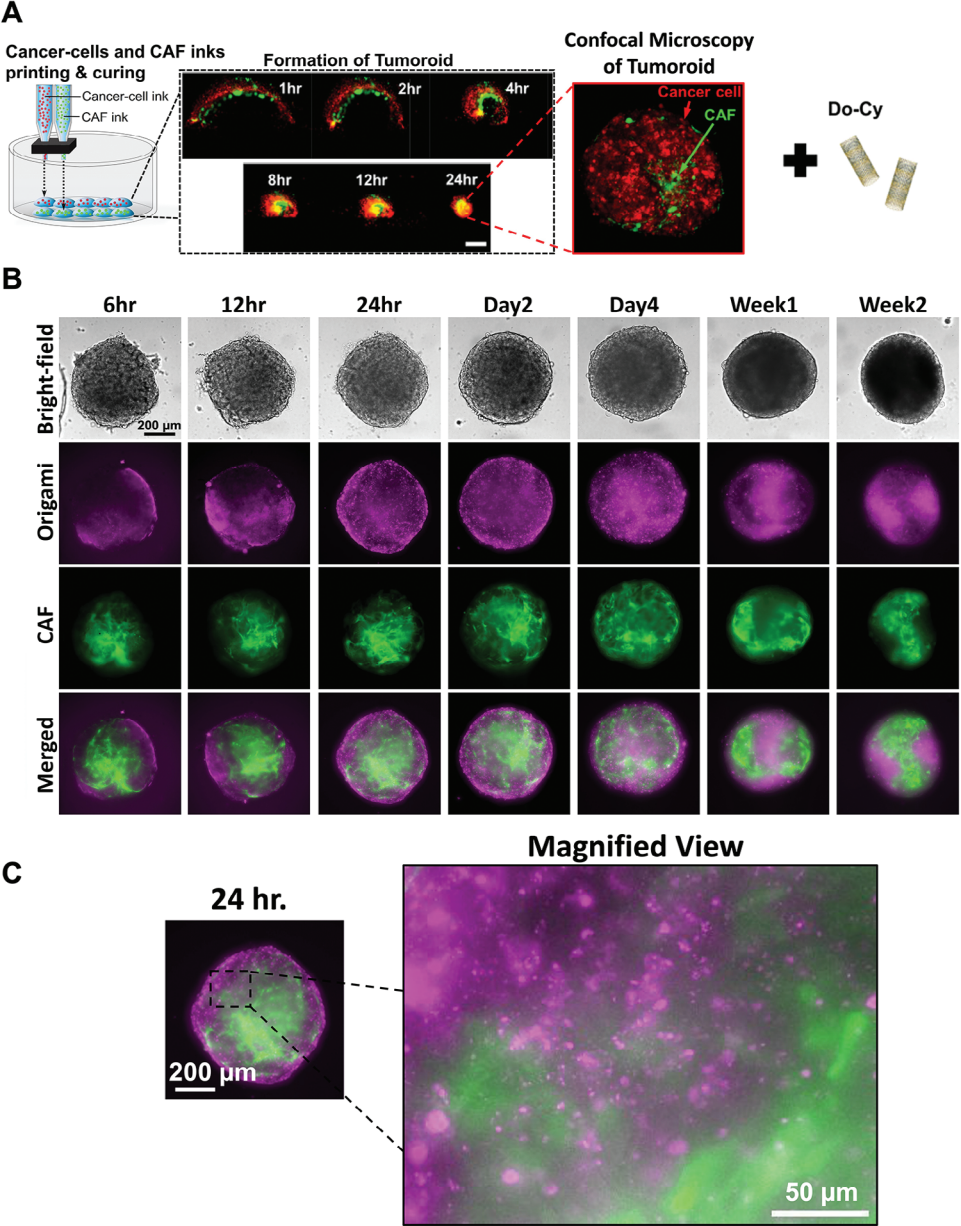
Tumor‐preferential accumulation and prolonged internalization of DNA origami‐Cy nanocomplex in the 3D pancreatic tumoroid model. A) Schematic of inkjet printing of cell‐laden interpenetrating‐polymer inks along with the hypothesized mechanism of tissue compaction. After tumoroid formation, Do‐Cy is added for long‐term culture. Confocal microscopy image demonstrates PCCs (red) forms on the outer edge of the tumoroid while CAFs (green) are present at the core. B) Bright‐field and fluorescent images showing the accumulation of Do‐Cy in the tumoroids at 6, 12, 24, 48, and 96 h, as well as 1 and 2 weeks. (magenta channel: Origami‐Cy, green channel: CAF). C) Magnified view at 24 h exhibits preferential uptake of Do‐Cy at non‐CAF locations, which is occupied by PCCs.

The tumoroid model comprised of Panc10.05 and CAF19 is developed through inkjet printed cell‐laden bioinks, the details of which were described in our prior study.^[^ ^59^ ^]^ Briefly, prepared cancer cell ink and CAF ink are printed and cured using the inkjet printing setup, as shown in the schematic process in **Figure** **6**A. Initially, five drops of cancer cell‐laden ink were deposited onto a glass well‐plate, creating a line array, and then cured for 1 min at 37 °C for IPI gelation. Subsequently, five drops of CAF‐laden ink were deposited adjacent to the first line and similarly cured for 1 min at 37 °C to gel the entire structure. Following this, cell medium was added, and the polymer matrix underwent compaction due to contractile forces generated by the cells. Ultimately, a 3D tumor‐stroma model, known as a tumoroid, is formed as the matrix compacted.

We examine the tumor preferential accumulation and prolonged internalization of Do‐Cy using 3D cell tumoroid models developed in our previous study.^[^ ^59^ ^]^ The 3D tumoroids are engineered to include pancreatic tumor cells and stroma components including CAFs. The tumoroid models feature a mechanically dynamic tumor–stroma interface at elevated cell density, achieved through the remodeling of a cell‐laden polymer matrix mediated by cellular contractile forces. As observed in Figure 6A, the confocal image of the tumoroid demonstrates spatial distribution of the two cell lines where CAFs are formed at the center of the tumoroid while cancer cells are present at the periphery due to differences in the contractile forces of the cells.

The design of these tumoroids allows us to more accurately mimic the complex microenvironment of pancreatic tumors. Specifically, the elevated cell density and dynamic remodeling create a realistic setting for studying the interactions between tumor cells and stromal components. Consistent with the observations from 2D in vitro models, we observe a tumor‐selective accumulation of Do‐Cy within cancer cells compared to CAFs as shown in Figure 6. This selective internalization is attributed to the elevated levels of macropinocytosis in KRAS‐mutant tumor cells, a mechanism less prominent in stromal cells.

To further validate our findings, we conducted a temporal analysis to assess the prolonged internalization of Do‐Cy in the tumoroids. As demonstrated in Figure 6B, fluorescence imaging over periods of 6, 12, 24, 48, and 96 h, as well as 1 and 2 weeks, revealed sustained accumulation of Do‐Cy in the outskirts of the tumoroid that is mainly occupied by the tumor cells as demonstrated in the confocal image, whereas fluorescence intensity in CAFs remained significantly lower throughout the study. The magnified view in Figure 6C more accurately demonstrates this. This prolonged internalization indicates that Do‐Cy not only preferentially targets tumor cells but also retains within these cells over extended periods, potentially enhancing the efficacy of imaging and therapeutic applications.

#### 2.4.2 PDAC Microphysiological System

**Figure 7:**
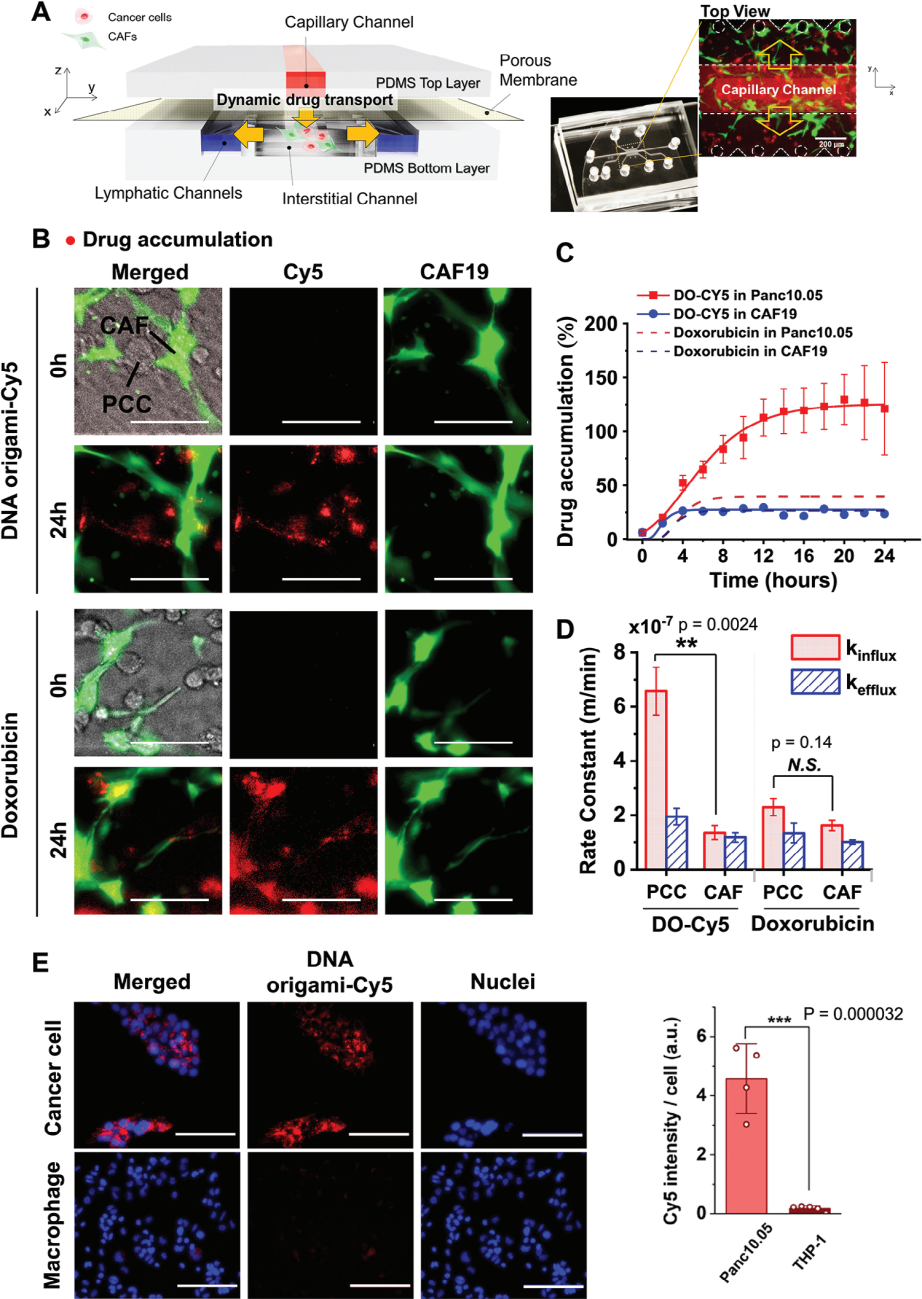
Quantitative evaluation for selective uptake of Do‐Cy in in vitro PDAC MPS model. A) Schematic configuration of the PDAC MPS model and its operation. B) Micrograph of the drug accumulation of Do‐Cy and doxorubicin (control for comparison^[^ ^102^ ^]^). Red represents accumulated Do‐Cy or doxorubicin, and green indicates the GFP‐transduced CAFs. C) Relative drug accumulation of Do‐Cy (solid lines) and doxorubicin (dashed lines) measured for cell type‐specific areas, compared with the mean intensity in capillary channels, respectively. Dots indicate Mean intensity ± S.D. (n = 3 each case). D) Quantified influx (K_influx_) and efflux (K_efflux_) affinity defined by cellular capability modeled by the mass conservation. Bars indicate mean ± S.E. (n = 3). P‐values of <0.05, <0.01, and <0.001 are represented as *, **, and ***, respectively, and *N.S*. indicates no statistical significance with P‐values ≥ 0.05 (Student t‐test). E) Do‐Cy accumulation in activated THP‐1. Bars indicate mean ± S.E., and dots represent each data point (n = 4). Scale bars indicate 100 µm.

Expanding our investigation into the quantitative approaches for the selective transport and accumulation of Do‐Cy in cancer cells, we used a microfluidic tumor microenvironment‐on‐a‐chip model (T‐MOC) developed in our previous study.^[^ ^60^, ^61^, ^62^ ^]^ The biomimetic T‐MOC model of PDAC stroma, as a PDAC microphysiological system (MPS), is comprised of pancreatic cancer cells and CAFs embedded in a dense type I collagen matrix. Moreover, the PDAC MPS recapitulates pharmacokinetic processes, generating fluid flow across an endothelium‐mimicking membrane interfaced with a capillary channel, mimicking extravasation from capillary vessels, interstitial diffusion and convection, cellular uptake, and lymphatic drainage.^[^ ^60^, ^63^, ^64^ ^]^ (**Figure** **7**A). In the T‐MOC model, we perfused Do‐Cy solutions along the capillary channel, monitoring transient drug accumulation in the cells using time‐lapse microscopy over 24 h. Consequently, the fluorescence images presented in Figure 7B display a significant accumulation of Do‐Cy (in red) within cancer cells (Panc10.05), with minimal fluorescence intensity detected within the regions occupied by green fluorescent CAFs (CAF19). In contrast, the doxorubicin drug, characterized by autofluorescence in red, is accumulated regardless of the cell type. The drug accumulation is further quantified according to the cell types (Figure 7C). The transient drug accumulation of Do‐Cy is notably enhanced within Panc10.05 compared with CAF19, allowing us to quantitatively measure the tumor‐targeting performance of Do‐Cy.

Furthermore, we estimate intracellular transport parameters specific to each cell type, by employing a transient drug accumulation model based on mass conservation principles as developed in our prior study.^[^ ^62^ ^]^ (see details in Supporting Information). In this model, the cell surface's drug influx affinity (k_influx_) reflects the cell's capability to uptake the drug, while the drug efflux affinity (k_efflux_) indicates the cell's capacity for drug outflow. We assume k_influx_ and k_efflux_ to be constant values representing the cellular capability for intracellular transport of the respective drugs. The results in Figure 7D demonstrate a significantly higher k_influx_ for PCC cells compared to CAF cells for Do‐Cy. Additionally, although the k_influx_ for doxorubicin in Panc10.05 cells surpassed that of CAF19 cells, the difference is less distinct compared to Do‐Cy, supporting the hypothesis that the selective uptake of Do‐Cy conjugate is triggered by cancer‐cell specific endocytic activity, such as macropinocytosis.

We further elucidate the accumulation patterns of Do‐Cy using the THP‐1 immune cell model to investigate its potential uptake in macrophages. THP‐1 monocytes and their phorbol ester (PMA) activated macrophages are reported to demonstrate elevated phagocytosis activity of various pathogens and nanoparticles and possess plasticity to be further differentiated to diverse tumor‐associated macrophage phenotypes.^[^ ^65^, ^66^, ^67^ ^]^ Using this approach, our observations indicate that Do‐Cy is not significantly captured by macrophages (Figure 7E). Additionally, the results suggest that our in vitro model effectively represents the accumulation of Do‐Cy in PCCs and CAFs, providing a reliable platform for accurate measurement.

#### 2.4.3 Tumor–Stroma Xenograft Model

To validate our in vitro findings and explore the distribution of Do‐Cy in more physiologically relevant conditions, we conducted an in vivo experiment using a xenograft mouse model. The mouse model is developed to feature human pancreatic tumor tissue, incorporating both pancreatic cancer cells and CAF cells to mimic the complex tumor microenvironment. To differentiate between these cell types, we utilize transfected CAF cells expressing GFP within the cytoplasm. Prior to administering Do‐Cy into the mouse model, we carefully monitor tumor growth post‐inoculation to establish the optimal proportion of cancer cells and CAF cells. Subsequently, Do‐Cy is delivered via tail vein injection on Day 28, which is determined as an optimal timepoint in which sufficient CAF population is present as confirmed by anti‐GFP staining (Figure S8, Supporting Information).

**Figure 8:**
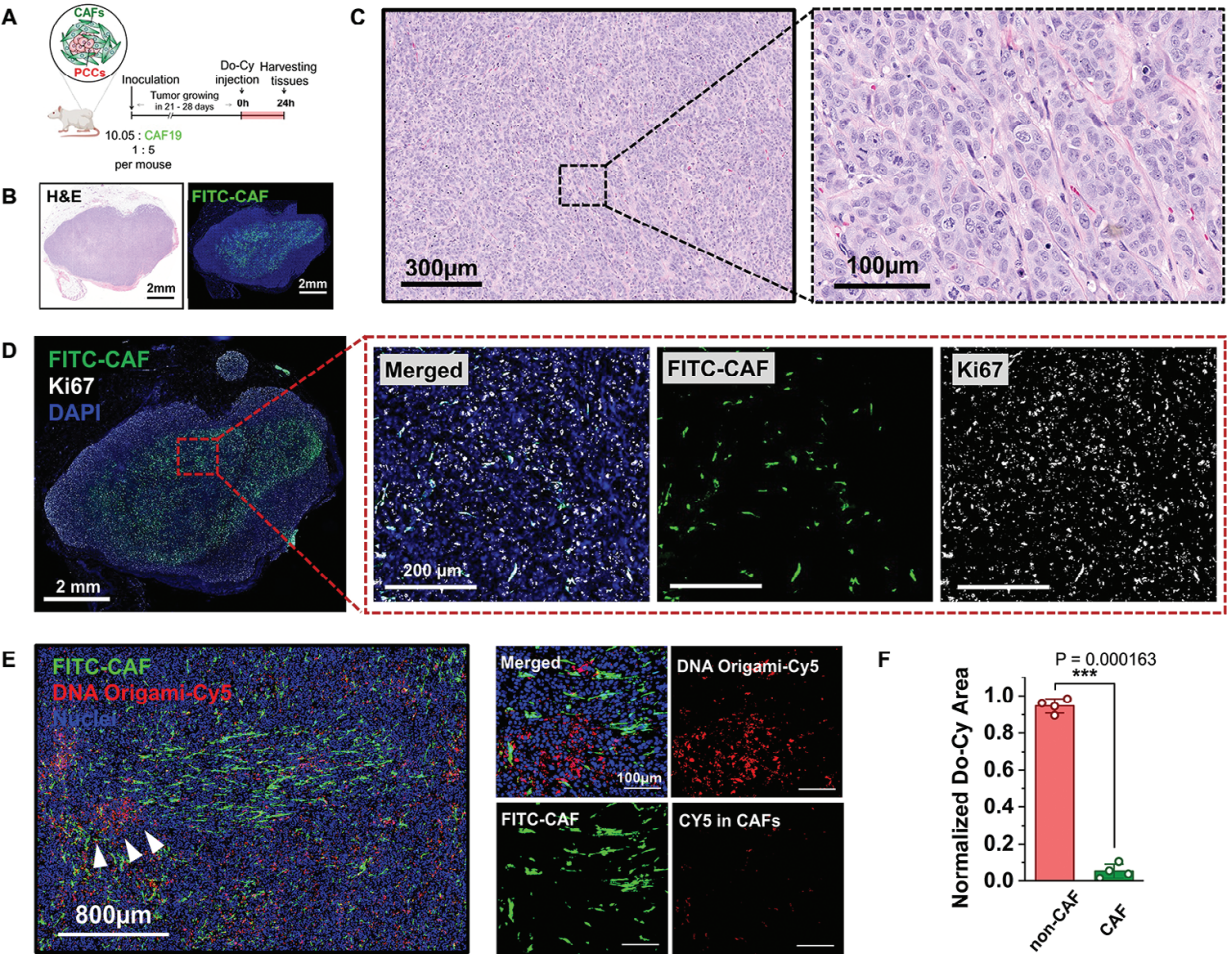
Xenograft mouse model for evaluation Do‐Cy in vivo distribution. A) Schematic illustration represents the PDAC stromal models inoculating PCC with CAFs with a ratio of 1:5. Detailed timeline for the experiment. B) Histological stain of hematoxylin plus eosin (H&E) and immunofluorescence image of the whole tumor tissue. Significant CAF population, indicated by GFP staining, is observed. The CAF population is also populated at the tumor core, consistent with observations in the tumoroid model. C) Magnified H&E image for detailed view of the PDAC and stroma composition. D) Ki67 staining shows significant expressions of proliferating cancer cells in non‐CAF regions. E) Immunofluorescence to visualize the distribution of Cy dye within the tumors by using anti‐Cy (red). The GFP‐CAF population is represented in green (FITC‐CAF). F) Quantification of Cy signal demonstrates that most of Do‐Cy signals are detected in non‐CAF cancer cell regions, suggesting selective uptake of Do‐Cy by the PCCs. Bars indicate mean ± S.E., and dots represent each data point (n = 4). P‐values of <0.05, <0.01, and <0.001 are presented as *, **, and ***, respectively (Student t‐test).

Following injection, Do‐Cy is allowed to circulate for 24 h before harvesting tumor tissues to capture the accumulation of Do‐Cy within the tumors. The 24 h time point is chosen to minimize potential interference of fluorescent signals from Do‐Cy in the bloodstream, thus ensuring that the fluorescence detected is primarily due to uptake by cells. Both H&E and immunostaining with anti‐GFP demonstrate development of tumor microenvironment with significant CAF population near the center of the tissue, similar to what is observed in in vitro tumoroids (**Figure** **8**B,C). Moreover, significant non‐GFP regions can be assumed as proliferating PCCs as evidenced by Ki67 staining that demonstrate considerable expression in non‐CAF regions (Figure 8D). As consistently observed in our in vitro models, the histological analysis demonstrates that higher concentrations of Do‐Cy accumulate in PCCs rather than CAFs, as illustrated in Figure 8E and quantification in Figure 8F. This trend mirrors our previous findings and reinforces the notion that Do‐Cy selectively accumulates in cancer cells over stromal cells within the TME. This trend mirrors our previous findings and reinforces the notion that Do‐Cy selectively accumulates in cancer cells over stromal cells within the TME.

## 3 Discussion

In this work, we utilized conjugated DNA origami tubules, known as Do‐Cy, for selectively imaging KRAS mutant pancreatic cancer cells. Our DNA nanostructure is designed with modularity such that its size (e.g., length) and shape can be changed and controlled. While we investigated 0.1X, 1X, and 2X lengths in our experiment, it has the potential to form 4X, 8X, and even larger polymers thus providing flexibility in design and experiment. The tubular shape also allows for considerable drug‐loading capacity due to a large internal cavity and facilitates the convenient design of docking points for imaging agents and drugs on the tube. This programmability and addressability would open up promising pharmaceutical applications for the future, improving delivery and pharmacokinetics of therapeutic molecules. In addition, there is an extensive library of fluorescent dyes that can be functionalized on DNA molecules. For example, fluorophores may be chosen from the entire visible spectrum and part of near infrared range covering from 400 to 800 nm.^[^ ^68^, ^69^ ^]^ Pairs of dye molecules may also be selected for Förster resonance energy transfer (FRET) measurement to probe molecular structures and dynamic processes in cancer cells.^[^ ^70^, ^71^ ^]^ Lastly, various ligands or other chemical modifications may be used to promote cellular uptake of DNA nanostructures. In our experiment, we refrained from such modifications, instead attaching fluorescent probes with strands on the origami to avoid disturbing the natural uptake pathways. Yet it is possible to enhance the DNA nanostructures by using affinity ligands for targeting specific locations or chemical moieties for suppressing enzymatic digestions.^[^ ^72^, ^73^, ^74^, ^75^ ^]^

Besides the size, it is possible that unique structural and chemical properties of Do‐Cy nanocomplex further enhance selective uptake.^[^ ^76^, ^77^ ^]^ Its natural nucleic acid composition provides biocompatibility and low toxicity, making DNA origami well‐suited for cellular interaction with minimal off‐target effects. While other nanoparticles with similar sizes could theoretically target cancer cells, they may lack the specific chemical properties of DNA origami that contribute to enhancing tumor‐specific targeting and reducing toxicity.^[^ ^78^ ^]^ We also hypothesize that the flexibility (or rigidity) of DNA structures may impact on the cellular uptake, which will be an interesting topic to investigate in future studies.

The present results suggest that Do‐Cy is preferentially internalized via elevated macropinocytosis by PCC over CAF. Macropinocytosis in cancer has been extensively studied to understand the molecular mechanisms and developing new imaging and therapeutic strategies targeting cancers.^[^ ^51^, ^79^, ^80^ ^]^ Macropinocytosis is thought to be associated with the Ras and PI3K signaling pathways in cancer cells by the activation of oncogenes including KRAS or deactivation of tumor suppressor genes, PTEN.^[^ ^80^, ^81^ ^]^ Since the membrane and cytoskeleton are involved in the process, molecular regulators of these processes are also identified, including small GTPase (Ras, Rac, Cdc42), p21‐activated kinase 1 (Pak1), and phosphoinositide 3‐kinase (PI3K).^[^ ^51^, ^81^, ^82^ ^]^ While growth factor signals stimulate macropinocytosis in normal cells, oncogenic cells have intrinsic constitutive macropinocytosis that has adapted to the nutrient scarce microenvironment. Macropinocytosis supports various metabolic pathways for cancer cell survival such as reductive carboxylation and glutamine oxidation, acetyl‐CoA cycle, and serine‐glycine cycling.^[^ ^79^, ^80^, ^83^, ^84^, ^85^ ^]^

Among these mechanisms, the present results of the elevated uptake of Do‐Cy by PCC are thought to be associated with KRAS‐dependent macropinocytosis. PDAC results from accumulated oncogenic mutations in ductal or acinar cells of the exocrine pancreas. PDAC tumorigenesis arises from acinar‐to‐ductal metaplasia (ADM) during which acinar cells undergo dedifferentiation into ductal‐ or epithelial‐like cells.^[^ ^86^ ^]^ Primordial lesions are characterized by intraepithelial neoplasias (PanIN; which is known to be the most common), mucinous cystic neoplasia (MCN), and intraductal pancreatic mucinous neoplasia (IPMN). During tumor development, the most frequent oncogenic mutations for PDAC are activation of *KRAS* mutations and inactivation of tumor suppressor genes such as *CDKN2A/p16*, *SMAD4*, and *TP53*.^[^ ^87^, ^88^, ^89^ ^]^ At the PDAC stages, activating mutations in KRAS are present in ≈100% of tumors. Inactivating mutations of CDKN2A and TP53 occur in 90% and 75% of pancreatic cancers, respectively.^[^ ^60^, ^90^, ^91^, ^92^ ^]^ KRAS mutation can occur in multiple variations of the codon 12, with G12C being the most common. Such variability may occur even within the same tumor leading to heterogeneity and chemoresistance. Recently, it has been identified that pancreatic cancer cells exert RAS dependent macropinocytosis for uptake of amino acids within the nutrient deficient TME despite variability of the RAS mutations.^[^ ^51^ ^]^ Moreover, additional evidence has demonstrated that PDAC cell lines with elevated KRAS mutation, such as MIA PaCa‐2, expressed increased macropinocytosis compared to cell lines without KRAS mutation, such as BxPC‐3.^[^ ^93^ ^]^ Various peptide‐conjugated drug delivery systems delivering conventional chemotherapy drugs and DNA PK inhibitors demonstrated enhanced macropinocytosis and drug accumulations in KRAS‐mutant tumors.^[^ ^51^, ^80^, ^94^ ^]^ Thus, the presently proposed precise delivery of Do‐Cy to PCC via KRAS‐dependent macropinocytosis is highly relevant for imaging PCC due to the prevalent KRAS mutations. This innovative approach opens new avenues for novel therapeutic strategies by leveraging the flexibility of the Do system's easy modification to deliver therapeutic agents.^[^ ^95^ ^]^ In this context, the analysis of selective accumulation of DNA origami within the PDAC tumor microenvironment holds a great promise in developing novel therapeutic applications.

While our study demonstrates that the Do‐Cy nanocomplex is preferentially internalized by KRAS‐mutant pancreatic cancer cells through elevated macropinocytosis, the underlying mechanism of macropinocytosis is not unique to PCCs. Oncogenic RAS mutations, which drive macropinocytosis, are also a known process in other cancer types, including non‐small cell lung cancer, colorectal cancer, and bladder cancers. Additionally, other pathways, including EGFR/HER2 signaling in breast cancer, can activate the RAS pathway, further hyperactivating macropinocytosis as a cancer‐specific uptake mechanism. This suggests that the Do‐Cy nanocomplex could potentially exhibit selective targeting in other KRAS‐mutant cancers. Indeed, studies have demonstrated cancer specificity in small lung cancer cells^[^ ^46^ ^]^ and breast cancer,^[^ ^21^ ^]^ although the underlying mechanisms have not been thoroughly investigated. To further validate this hypothesis, additional studies are warranted to evaluate the uptake and specificity of Do‐Cy nanocomplexes in the other cancer cell types known to show hyperactivated macropinocytosis. Such investigations would clarify whether the DNA origami is a PCC‐specific structure or broadly applicable to other cancer types.

Although the present results indicate macropinocytosis as the primary uptake mechanism in KRAS‐mutant PDAC cells, alternative endocytic pathways may contribute to the uptake of Do‐Cy nanostructures in other cell types. Many studies have highlighted the cell‐type dependency of nanostructure responses, underscoring the importance of quantitative and comprehensive evaluations of nanostructure delivery mechanisms.^[^ ^50^, ^96^ ^]^ BxPC‐3 cells without oncogenic KRAS mutations, still exhibited higher accumulation of Do‐Cy (Figure S9, Supporting Information). Interestingly, their uptake was significantly reduced by both the macropinocytosis inhibitor (EIPA) and the clathrin‐mediated endocytosis inhibitor (chlorpromazine), suggesting the involvement of distinct or overlapping endocytic pathways. Further investigation is needed to explore these potential variations.

Given our objective of developing DNA origami‐based nanomaterials that enable selective targeting of pancreatic cancer cells with minimal accumulation in other stromal cells in TME, our study highlights the critical role of nanostructure size in achieving this specificity. The size of nanostructures plays a crucial role in their interaction with cellular uptake mechanisms, potentially by aligning with the physiological constraints of endocytic vesicles.^[^ ^96^ ^]^ The optimal size of tubular Do‐Cy nanocomplex in the study, with dimensions of ≈70 nm in length and 30 nm in diameter, falls within the size range typically internalized by both macropinocytosis and clathrin‐mediated endocytosis.^[^ ^96^, ^97^ ^]^ However, our results indicate that macropinocytosis is the primary pathway for uptake of KRAS mutant PDAC cells, as evidenced by significant reductions in internalization upon the use of macropinocytosis inhibitors, whereas clathrin‐mediated endocytosis inhibitors did not alter uptake. This confirms macropinocytosis as the primary mechanism driving the differential cellular uptake of these DNA origami nanostructures in PDAC. Our findings emphasize the need for quantitative evaluations to determine the optimal size range for effective targeting. While further research is needed to elucidate the precise mechanisms underlying the size dependency of Do‐Cy uptake, such evaluations are essential for achieving size‐specificity in the selective uptake of nanostructures by pancreatic cancer cells while minimizing accumulation in other stromal cells.

Additionally, while shape has been associated with organ‐specific biodistribution in prior studies,^[^ ^50^ ^]^ our findings suggest it has a minimal impact on cellular uptake within the TME. Prior in vivo studies have demonstrated that nanostructure shape can influence circulation time, biodistribution, and organ‐specific uptake, with distinct preferences emerging based on physiological and anatomical barriers. However, within the TME, the influence of shape appears to be minimized by the predominant uptake pathways, such as macropinocytosis, which is less selective regarding particle morphology. This study highlights the limitations of relying on shape as a determinant for specificity in the TME and highlights the necessity for precise, quantitative evaluations of DNA nanostructure design to achieve optimal therapeutic outcomes.

The systematic evaluation of the cancer‐targeting drug delivery of Do‐Cy using various in vitro and in vivo models with the inclusion of CAFs demonstrated efficient platforms to evaluate tumor‐selective accumulation. Our analysis of Do‐Cy transport characteristics, which includes the interactions between cancer cells and CAFs, is conducted across conventional in vitro, MPS, and in vivo models. Our quantitative assessment of the accumulation of Do‐Cy5 in cancer cells within various microphysiological systems supports our initial hypothesis that cancer‐cell specific macropinocytosis activity enhances the uptake of nanocomplex selectively in PDAC cells compared to CAFs. Specifically, in the MPS based analysis we observed a significantly higher uptake affinity compared to efflux affinity, indicating an upregulation of intake by cancer cells facilitated by macropinocytosis. The consistent results across multiple platforms highlight the promising potential of the engineered human‐mimicking in vitro system as a next‐generation technology that could complement existing preclinical in vivo mouse studies, especially considering recent shifts in the FDA regulations. This systematic evaluation pipeline provides detailed quantitative information on the intricate dynamics of tumor‐selective accumulation and offers rapid discovery and evaluation of innovative therapeutic strategies.

## 4 Experimental Section

### DNA Origami Synthesis

All DNA origami nanostructures were assembled from the same m13mp18 DNA scaffold purchased from Bayou Biolabs. All staple oligonucleotides and Cy5 modified strands were purchased from Integrated DNA Technologies. The sequence information is provided in Tables S1 through S5 (Supporting Information).

The 1X and 0.1X DNA tubes were prepared by following the same procedure of mixing 10 nm scaffold strands and 4x DNA staples in 1x TAE buffer (40 mm trisaminomethane, 1 mm ethylenediaminetetraacetic acid (EDTA) disodium salt, and 20 mm acetic acid, pH ≈8) with 12.5 mm Mg^2+^. The mixture was annealed from 75 to 25 °C at a rate of −1°C min^−1^ in a BIO‐RAD S1000 Thermal Cycler. The sample was then purified with an Amicon centrifugal filter (100 kDa) at 5000 RPM for 3 min to remove excess staple strands. Then, the Cy5 modified strands were added to the mixture at a concentration of 300 nm and kept at 40 °C for an hour. Finally, the mixture was purified again with Amicon centrifugal filter (100 kDa) at 5000 RPM for 3 min to remove excess Cy5‐strands. The final origami sample was used for characterizations and experiments.

The 2X origami was prepared by combining two 1X origami tubules. Tubular origami A and B were used to form a dimer origami with a length of ≈140 nm by stacking each other. Tube A is the 1X origami and was prepared with Cy5 dyes as described above. Tube B uses a different set of staples and was prepared without Cy5 modified strands. The two types of DNA tubes were prepared separately and then mixed together with ‘linker’ strands at 500 nm that connect A and B tubes. The mixture was kept overnight at room temperature to allow dimerization and then was purified again with Amicon centrifugal filter (100 kDa) at 5000 RPM for 3 min to remove excess linkers. The final product was dimers with twice in length.

### Cells and Reagents

Human pancreatic cancer cells (Panc10.05, MIA PaCa‐2, Panc1) and cancer‐associated fibroblasts (CAF19 and CAF02) were maintained in Advanced Dulbecco's Modified Eagle's Medium/Ham's F‐12 (DMEM/F12, Invitrogen, NY, USA) with 2.05 mm L‐glutamine (GE Healthcare Bio‐Sciences Corp., MA, USA) supplemented by 5% v/v fetal bovine serum (FBS) and 100 µg mL^−1^ penicillin/streptomycin (P/S). The cells were regularly harvested by 0.05% trypsin and 0.53 mm EDTA (Life Technologies, CA, USA) when grown to ≈80% confluency in 75 cm^2^ T‐flasks and incubated at 37 °C with 5% CO_2_. Harvested cells were used for experiments by culturing on 2D and iT‐MOC, or sub‐cultured while maintaining them below 15th passage.^[^ ^98^ ^]^

The KRAS‐inducible human pancreatic ductal epithelial cells (HPDE iKRAS, provided by Dr. Brittney Allen–Peterson at Purdue University) cell line was genetically modified to allow for the KRAS G12D mutation to be induced by the presence of doxycycline.^[^ ^99^ ^]^ The modification and characterization of the cell line are described in Tsang et al.^[^ ^55^ ^]^ HPDE iKRAS cells were maintained in Keratinocyte‐Serum Free medium (Invitrogen, MA, USA) supplemented by Bovine Pituitary Extract (0.05mg mL^−1^), recombinant human epidermal growth factor (5ng mL^−1^) and L‐Glutamine.

For KRAS induction, HPDE iKRAS cells were treated with 25ng mL^−1^ doxycycline for 48 h (HPDE KRAS^mut^), whereas the HPDE iKRAS cells were treated with a corresponding DMSO control media for HPDE KRAS^wt^ in the normal culture conditions. The KRAS induction was processed after the cells were harvested and seeded in the experimental platforms.

THP‐1 cells were kept in culture in complete RPMI media supplemented with 10% FBS, 1% MEM non‐essential amino acids (Thermo Fisher Scientific, MA, USA), 1% sodium pyruvate, 1% HEPES (Life Technologies, CA, USA), and 1% P/S. Cells were seeded in 96 wells at 5000 cells/well density and treated with 10 ng mL^−1^ phorbol 12‐myristate 13‐acetate (PMA, Sigma–Aldrich, MO, USA) for 24 h. Subsequently, wells were washed with complete RPMI media and cellular uptake assay was performed as previously explained.

### Cellular uptake Assay

The cells were seeded on 96 well plates at the density of 2000 cells/well and then pre‐cultured in normal media for at least 48 h before performing the cellular uptake assay. For the inhibitor treatment experiment, cells were pre‐incubated with 5‐(N‐Ethyl‐N‐isopropyl)amiloride (EIPA,50 µm) or Chlorpromzine, 20 µm for 1.5 h before exposing DO‐Cy5. The control groups were pre‐incubated with DMSO. Afterward, the cells were exposed to DO‐Cy5 for 24 h. At the 24 h time point, the nuclei were stained with Hoechst 33342 (Thermo Fisher, Waltham, MA). Fluorescence images were obtained at various time points between 0 and 24 h using an Olympus IX71 inverted microscope.

### 3D Tumoroids Model

The tumoroid model comprised of Panc10.05 and CAF19 was developed through inkjet printed cell‐laden bioinks, details were described in the prior study.^[^ ^59^ ^]^ Briefly, prepared cancer cell ink and CAF ink are printed and cured using the inkjet printing setup. Initially, five drops of cancer cell‐laden ink were deposited onto a glass well‐plate, creating a line array, and then cured for 1 min at 37 °C for IPI gelation. Subsequently, five drops of CAF‐laden ink were deposited adjacent to the first line and similarly cured for 1 min at 37 °C to gel the entire structure. Following this, cell medium was added, and the polymer matrix underwent compaction due to contractile forces generated by the cells. Ultimately, a 3D tumor‐stroma model, known as a tumoroid, was formed as the matrix compacted.

### 3D PDAC MPS Model

The accumulation pattern and uptake of DO‐Cy5 in PDAC were assessed using the PCC‐CAF co‐cultured T‐MOC model, developed as a 3D PDAC MPS model to mimic the PDAC stroma. Specifically, the transport and accumulation were investigated to reveal distinct responses between PCC and CAF cells in DO‐Cy5 transport under dynamic tumor microenvironmental conditions. The PDAC MPS model is a microfluidic platform designed to demonstrate dynamic transport, as shown in the prior studies,^[^ ^60^, ^63^, ^64^ ^]^ comprising capillary, interstitial, and lymphatic channels. In the model, drug transport is simulated under physiological conditions, passing from capillary through interstitial and lymphatic channels. To achieve this, a hydrostatic pressure difference of 20 mm H_2_O was used to demonstrate the average interstitial flow rates observed in the TME.^[^ ^100^ ^]^ The drug solution (30nm of DO‐Cy5 or doxorubicin) was perfused through the capillary channels, exposed the conditions for 24 h, and measured the accumulation patterns of DO‐Cy5 transiently by taking the fluorescence intensity of DO‐Cy5/doxorubicin every 2 h using a live‐cell imaging technique with time‐lapse microscopy. An inverted microscope (Olympus IX71, Japan) was equipped with the stage top incubator to maintain the culture conditions at 37 °C with 5% CO_2_ during imaging.^[^ ^101^ ^]^

Temporal drug accumulation in the cells was measured by fluorescence intensity at each cell type where the intensity was calibrated with fluorescence of 30 nm of the corresponding compound. The accumulation was measured for each cell type separately. To ensure accurate measurements and avoid misclassification between cell types, CAF regions were specifically excluded from the analysis of PCC regions, with PCC areas identified based on their distinct morphology (Supporting Information). The control experiment with doxorubicin also followed the above procedure to compare the differential drug accumulation. All experiments were repeated at least three times for each treatment group. The data was reported in the form of mean ± standard deviation.

### Xenograft Model

Panc10.05 and CAF19 cells were cultured for subcutaneous model development of xenograft mice. For subcutaneous injections, cells were prepared in 1:5 PCC:CAF ratio (2×10^6^: 10×10^6^ cells per mouse) were prepared in 100 µL sterile PBS and mixed with 100 µL Matrigel for implantation into NRG mice. After 3 weeks, DNA origami was delivered via tail vein injection 200 µL mouse^−1^. After 24 h of origami injection, tumors were harvested and formalin‐fixed at the end of the experiment for further histology analysis. The experiment was replicated with four mice.

### Histology

For paraffin sections, tissues were fixed in 10% formalin. Subsequently, they were subjected to dehydration using graded ethanols, followed by clearing in xylene and infiltration with Leica Paraplast Plus paraffin using a Sakura Tissue‐Tek VIP6 tissue processor. Following processing, the tissues were embedded in Leica Paraplast Plus paraffin. H&E staining was performed using the Leica Autostainer XL, the slides are stained using Gill's II hematoxylin, then blued and counterstained with a mixture of eosin and phloxine B. Subsequently, they undergo dehydration, clearing in xylene, and are finally cover‐slipped using a toluene‐based mounting medium (Leica MM24).

For immunofluorescence, slides were incubated in 2.5% normal horse serum for 20 min. Then, primary antibodies applied accordingly. Anti‐GFP was applied at 1:100 and anti‐Cy5 at 1:100 for 1 h. The negative control slide was stained with Rabbit IgG (Vector Labs, I‐1000) at a concentration of 1µg mL^−1^ for 1 h. Slides were rinsed twice in TBST and anti‐goat Alexa 555 secondary (Invitrogen, A21432) applied at 4 µg mL^−1^ for 30 min. An anti‐rabbit dylight 488 (Vector Labs, DI‐1488) was then applied at 6µg mL^−1^ for 30 min. Slides were rinsed in TBST and counterstained with DAPI (Invitrogen, EN62248) at 1 µg mL^−1^ for 10 min before rinsing and coverslipping with Prolong Gold (Invitrogen, P36934). All images were taken using a Leica Versa8 whole‐slide scanner.

For Ki67 staining, slides were deparaffinized and hydrated as three times in Xylene, 3 min each time; two times in 100% alcohol, 1 min each time; 1 min in both 95% alcohol and 70% alcohol; then rinsed with distilled water and transferred to PBS for 5 min. Slides were then heated at 95°C for 20 min in citrate buffer, followed by cool at room temperature and washed with PBS. Slides were then blocked with 1% BSA and primary antibody applied as Cy5 (Sigma–Aldrich, C1117) at 1:150, GFP (Bio‐Techne, AF4240) at 1:100 and Ki67/MKI67 (Bio‐Techne, MAB7617) at 1:100 overnight at 4C. After three times wash with PBST for 3 min each time, secondary antibodies applied as Donkey anti‐Mouse Alexa 488 (Invitrogen, A21202) at 1:200, Donkey anti‐Goat Alexa 594 (Invitrogen, A11058) and Donkey anti‐Rabbit Alexa 680 (Invitrogen, A21202) at 1:100 for 2 h at room temperature. Slides were then washed three times with PBST for 3 min each time and counterstained with DAPI (Invitrogen, H3570) at 1 µg mL^−1^ for 10 min before rinsing and covers lipping with Micromount (Leica, 3801731). Slides were scanned with PhenoImager (Akoya).

### DNA Origami Stability Test

Pancreatic cancer cells were lysed using Genomic Lysis Buffer in Quick‐DNA Microprep Kit (Zymo Research, Irvine, CA). The Do‐Cy samples incubated in each cell lysate were analyzed by electrophoresis in 1.0% agarose gel containing 0.5x TBE and 11 mm MgCl₂. Electrophoresis was carried out for 2–3 h at a constant voltage of 75 V in a gel box immersed in a water bath. 10x TBE buffer (89 mm Tris, 89 mm boric acid, 2 mm EDTA disodium salt, pH ≈8.3), purchased from ThermoFisher, was diluted to 0.5x. The temperature was maintained below 35 °C using an ice pack. Target bands from each cell lysate, aligned with the position of the band of the prepared DNA origami, were carefully extracted using a razor and centrifuged with Freeze ‘N Squeeze tubes (BIO‐RAD, Hercules, CA) at 13 000g for 3 min. The recovered samples were then analyzed by AFM imaging.

### Statistical Analysis

All experiments were repeated at least three times for each group. The data was reported in the form of mean ± standard estimated error (S.E.) or mean ± standard deviation (S.D.), which is indicated respectively. Data points were statistically analyzed by using a student t‐test using software OriginPro 2021 (OriginLab Corporation, Northampton, MA). The statistical significance was represented by p‐value (p<0.05).

## Conflict of Interest

The authors declare no conflict of interest.

## Supporting information



Supporting Information

## Data Availability

The data that support the findings of this study are available from the corresponding author upon reasonable request.

## References

[1] R. L. Siegel , A. N. Giaquinto , A. Jemal , Ca‐Cancer J. Clin. 2024, 74, 12. 38230766 10.3322/caac.21820

[2] C. R. Drifka , J. Tod , A. G. Loeffler , Y. Liu , G. J. Thomas , K. W. Eliceiri , W. J. Kao , Mod. Pathol. 2015, 28, 1470. 26336888 10.1038/modpathol.2015.97

[3] A. Neesse , P. Michl , K. K. Frese , C. Feig , N. Cook , M. A. Jacobetz , M. P. Lolkema , M. Buchholz , K. P. Olive , T. M. Gress , D. A. Tuveson , Stromal Biology and Therapy in Pancreatic Cancer, Gut 2011, 60, 861. 20966025 10.1136/gut.2010.226092

[4] M. Erkan , S. Hausmann , C. W. Michalski , A. A. Fingerle , M. Dobritz , J. Kleeff , H. Friess , Nat. Rev. Gastroenterol. Hepatol. 2012, 9, 454. 22710569 10.1038/nrgastro.2012.115

[5] J. P. Morris , S. C. Wang , M. Hebrok , K. Hedgehog , Nat. Rev. Cancer 2010, 10, 683. 20814421 10.1038/nrc2899 PMC4085546

[6] E. Cukierman , D. E. Bassi , Seminars in Cancer Biol. 2010, 20, 139. 10.1016/j.semcancer.2010.04.004 PMC2941524 20452434

[7] J. T. Erler , V. M. Weaver , Clin Exp. Metastasis 2009, 26, 35. 18814043 10.1007/s10585-008-9209-8 PMC2648515

[8] C. J. Whatcott , C. H. Diep , P. Jiang , A. Watanabe , J. LoBello , C. Sima , G. Hostetter , H. M. Shepard , D. D. V. Hoff , H. Han , Clin Cancer Res. 2015, 21, 3561. 25695692 10.1158/1078-0432.CCR-14-1051 PMC4526394

[9] K. Cho , Y. Matsuda , J. Ueda , E. Uchida , Z. Naito , T. Ishiwata , Int. J. Oncol. 2012, 40, 1040. 22159401 10.3892/ijo.2011.1280 PMC3584520

[10] S. Vosseler , W. Lederle , K. Airola , E. Obermueller , N. E. Fusenig , M. M. Mueller , Int. J. Cancer 2009, 125, 2296. 19610062 10.1002/ijc.24589

[11] P. Zigrino , I. Kuhn , T. Bäuerle , J. Zamek , J. W. Fox , S. Neumann , A. Licht , M. Schorpp‐Kistner , P. Angel , C. Mauch , J. Invest Dermatol. 2009, 129, 2686. 19516266 10.1038/jid.2009.130

[12] M. Schober , R. Jesenofsky , R. Faissner , C. Weidenauer , W. Hagmann , P. Michl , R. L. Heuchel , S. L. Haas , J. M. Löhr , Cancers 2014, 6, 2137. 25337831 10.3390/cancers6042137 PMC4276960

[13] M. E. Fiori , S. Di Franco , L. Villanova , P. Bianca , G. Stassi , R. De Maria , Mol. Can. 2019, 18, 1. 10.1186/s12943-019-0994-2 PMC6441236 30927908

[14] C. Kaltenmeier , I. Nassour , R. S. Hoehn , S. Khan , A. Althans , D. A. Geller , A. Paniccia , A. Zureikat , S. Tohme , J. Gastro. Surgery 2021, 25, 2307. 10.1007/s11605-020-04870-6 PMC8169716 33269460

[15] W. S. Tummers , J. V. Groen , B. G. Sibinga Mulder , A. Farina‐Sarasqueta , J. Morreau , H. Putter , C. J. Van De Velde , A. L. Vahrmeijer , B. A. Bonsing , J. S. Mieog , R. J. Swijnenburg , British J. Surgery 2019, 106, 1055. 10.1002/bjs.11115 PMC6617755 30883699

[16] C. S. Verbeke , K. V. Menon , HPB 2009, 11, 282. 19718354 10.1111/j.1477-2574.2009.00055.x PMC2727080

[17] T. M. Lwin , R. M. Hoffman , M. Bouvet , Expert Rev. Anticancer Therapy 2018, 18, 651. 10.1080/14737140.2018.1477593 PMC6298876 29768067

[18] G. Lu , N. S. vanden Berg , B. A. Martin , N. Nishio , Z. P. Hart , S. van Keulen , S. Fakurnejad , S. U. Chirita , R. C. Raymundo , G. Yi , Q. Zhou , G. A. Fisher , E. L. Rosenthal , G. A. Poultsides , Lancet Gastro. Hepatol. 2020, 5, 753. 10.1016/S2468-1253(20)30088-1 PMC7367758 32416764

[19] L. Liu , P. G. Kshirsagar , S. K. Gautam , M. Gulati , E. I. Wafa , J. C. Christiansen , B. M. White , S. K. Mallapragada , M. J. Wannemuehler , S. Kumar , Theranostics 2022, 12, 1030. 35154473 10.7150/thno.64805 PMC8771545

[20] Q. Hu , H. Li , L. Wang , H. Gu , C. Fan , Chemical Rev. 2018, 119, 6459. 10.1021/acs.chemrev.7b00663 29465222

[21] Q. Zhang , Q. Jiang , N. Li , L. Dai , Q. Liu , L. Song , J. Wang , Y. Li , J. Tian , B. Ding , ACS Nano 2014, 8, 6633. 24963790 10.1021/nn502058j

[22] M. A. Dobrovolskaia , M. Bathe , Nanomed. Nanobiotechnol. 2021, 13, e1657. 10.1002/wnan.1657 PMC7736207 32672007

[23] P. Tanner , P. Baumann , R. Enea , O. Onaca , C. Palivan , W. Meier , Accounts of Chem. Res. 2011, 44, 1039. 10.1021/ar200036k 21608994

[24] F. Ye , Å. Barrefelt , H. Asem , M. Abedi‐Valugerdi , I. El‐Serafi , M. Saghafian , K. Abu‐Salah , S. Alrokayan , M. Muhammed , M. Hassan , Biomaterials 2014, 35, 3885. 24495486 10.1016/j.biomaterials.2014.01.041

[25] X. Guo , F. C. Szoka , Accounts of Chem. Res. 2003, 36, 335. 10.1021/ar9703241 12755643

[26] M. Maeki , N. Kimura , Y. Sato , H. Harashima , M. Tokeshi , Adv. Drug Del. Rev. 2018, 128, 84. 10.1016/j.addr.2018.03.008 29567396

[27] W. Paul , C. P. Sharma , Inorg. Nanoparticles for targeted drug del., Bioint. Med. Implant Mater. 2020, 333.

[28] A. Y. Cai , Y. J. Zhu , C. Qi , Adv. Mater. Inter. 2020, 7, 2000819.

[29] P. W. Rothemund , Nature 2006, 440, 297. 16541064 10.1038/nature04586

[30] Y. Du , J. Pan , J. H. Choi , Methods and Appl. Fluor. 2019, 7, 012002. 10.1088/2050-6120/aaed11 30523978

[31] R. Li , A. Madhavacharyula , Y. Du , H. Adepu , J. H. Choi , Chem. Sci. 2023, 14, 8018. 37538812 10.1039/d3sc01793a PMC10395309

[32] G. Tikhomirov , P. Petersen , L. Qian , Nature 2017, 552, 67. 29219965 10.1038/nature24655

[33] R. Iinuma , Y. Ke , R. Jungmann , T. Schlichthaerle , J. B. Woehrstein , P. Yin , Science 2014, 344, 65. 24625926 10.1126/science.1250944 PMC4153385

[34] S. Dey , C. Fan , K. V. Gothelf , J. Li , C. Lin , L. Liu , N. Liu , M. A. Nijenhuis , B. Saccà , F. C. Simmel , Nat. Rev. Methods Pri. 2021, 1, 13.

[35] Q. Jiang , S. Liu , J. Liu , Z. G. Wang , B. Ding , Adv. Mater. 2019, 31, 1804785. 10.1002/adma.201804785 30285296

[36] F. Li , T.‐G. Cha , J. Pan , A. Ozcelikkale , B. Han , J. H. Choi , ChemBioChem 2016, 17, 1138. 27059426 10.1002/cbic.201600052 PMC5051347

[37] Y. Wu , K. Sefah , H. Liu , R. Wang , W. Tan , Proc. Natl. Acad. Sci. USA 2010, 107, 5. 20080797 10.1073/pnas.0909611107 PMC2806697

[38] Y. Lu , J. Liu , Curr. Opin. Biotechnol. 2006, 17, 580. 17056247 10.1016/j.copbio.2006.10.004

[39] Q. Jiang , C. Song , J. Nangreave , X. Liu , L. Lin , D. Qiu , Z.‐G. Wang , G. Zou , X. Liang , H. Yan , J. American Chem. Soc. 2012, 134, 13396. 10.1021/ja304263n 22803823

[40] N. Plongthongkum , D. H. Diep , K. Zhang , Nat. Rev. Genetics 2014, 15, 647. 25159599 10.1038/nrg3772

[41] M. Meselson , R. YuAN , J. Heywood , Annual Rev. Biochem. 1972, 41, 447. 4563439 10.1146/annurev.bi.41.070172.002311

[42] D. Han , S. Pal , J. Nangreave , Z. Deng , Y. Liu , H. Yan , Science 2011, 332, 342. 21493857 10.1126/science.1202998

[43] H. Chen , T. G. Cha , J. Pan , J. H. Choi , Nanotechnology 2013, 24, 435601. 24076521 10.1088/0957-4484/24/43/435601

[44] Q. Mei , X. Wei , F. Su , Y. Liu , C. Youngbull , R. Johnson , S. Lindsay , H. Yan , D. Meldrum , Nano Lett. 2011, 11, 1477. 21366226 10.1021/nl1040836 PMC3319871

[45] K.‐R. Kim , D.‐R. Kim , T. Lee , J. Y. Yhee , B.‐S. Kim , I. C. Kwon , D.‐R. Ahn , Chem. Commun. 2013, 9, 2010. 10.1039/c3cc38693g 23380739

[46] P. Wang , M. A. Rahman , Z. Zhao , K. Weiss , C. Zhang , Z. Chen , S. J. Hurwitz , Z. G. Chen , D. M. Shin , Y. Ke , J. American Chem. Soc. 2018, 140, 2478. 10.1021/jacs.7b09024 PMC7261494 29406750

[47] T. Maezawa , S. Ohtsuki , K. Hidaka , H. Sugiyama , M. Endo , Y. Takahashi , Y. Takakura , M. Nishikawa , Nanoscale 2020, 12, 14818. 32633313 10.1039/d0nr02361b

[48] M. M. Bastings , F. M. Anastassacos , N. Ponnuswamy , F. G. Leifer , G. Cuneo , C. Lin , D. E. Ingber , J. H. Ryu , W. M. Shih , Nano Lett. 2018, 18, 3557. 29756442 10.1021/acs.nanolett.8b00660

[49] Y. Wang , E. Benson , F. Fördős , M. Lolaico , I. Baars , T. Fang , A. I. Teixeira , B. Högberg , Adv. Mater. 2021, 33, 2008457. 34096116 10.1002/adma.202008457 PMC7613750

[50] A. Rajwar , S. R. Shetty , P. Vaswani , V. Morya , A. Barai , S. Sen , M. Sonawane , D. Bhatia , ACS Nano 2022, 16, 10496. 35715010 10.1021/acsnano.2c01382

[51] H. Liu , F. Qian , Theranostics 2022, 12, 1321. 35154489 10.7150/thno.67889 PMC8771556

[52] B. Kim , Y. S. Park , J. S. Sung , J. W. Lee , S. B. Lee , Y. H. Kim , Cancer Med. 2021, 10, 372. 33314735 10.1002/cam4.3635 PMC7826488

[53] S. R. Elkin , N. Bendris , C. R. Reis , Y. Zhou , Y. Xie , K. E. Huffman , J. D. Minna , S. L. Schmid , Cancer Res. 2015, 75, 4640. 26359453 10.1158/0008-5472.CAN-15-0939 PMC4802864

[54] N. I. Marín‐Ramos , S. Ortega‐Gutiérrez , M. L. López‐Rodríguez , Seminars in cancer Biol. 2019, 54, 91. 10.1016/j.semcancer.2018.01.017 29409706

[55] Y. H. Tsang , T. Dogruluk , P. M. Tedeschi , J. Wardwell‐Ozgo , H. Lu , M. Espitia , N. Nair , R. Minelli , Z. Chong , F. Chen , Nat. Commun. 2016, 7, 10500. 26806015 10.1038/ncomms10500 PMC4737758

[56] M. C. Kerr , R. D. Teasdale , Traffic 2009, 10, 364. 19192253 10.1111/j.1600-0854.2009.00878.x

[57] D. Mathur , A. R. Galvan , C. M. Green , K. Liu , I. L. Medintz , Nanoscale 2023, 15, 2516. 36722508 10.1039/d2nr05868e PMC10407680

[58] L.‐j. Wang , M. Ren , L. Liang , C.‐y. Zhang , Chem. Sci. 2018, 9, 4942. 29938021 10.1039/c8sc01641k PMC5994793

[59] C. Cheng , N. Deneke , H.‐r. Moon , S. R. Choi , N. Ospina‐Muñoz , B. D. Elzey , C. S. Davis , G. T.‐C. Chiu , B. Han , Mater. Today Adv. 2023, 19, 100408. 37691883 10.1016/j.mtadv.2023.100408 PMC10486313

[60] H.‐r. Moon , A. Ozcelikkale , Y. Yang , B. D. Elzey , S. F. Konieczny , B. Han , Lab Chip 2020, 20, 3720. 32909573 10.1039/d0lc00707b PMC9178523

[61] A. Ozcelikkale , K. Shin , V. Noe‐Kim , B. D. Elzey , Z. Dong , J. T. Zhang , K. Kim , I. C. Kwon , K. Park , B. Han , J. Control Rel. 2017, 266, 129. 10.1016/j.jconrel.2017.09.024 PMC5723544 28939108

[62] K. Shin , B. S. Klosterhoff , B. Han , Mol. Pharmaceutics 2016, 13, 2214. 10.1021/acs.molpharmaceut.6b00131 PMC5032827 27228477

[63] A. Ozcelikkale , H. r. Moon , M. Linnes , B. Han , Nanomed. Nanobiotechnol. 2017, 9, e1460. 10.1002/wnan.1460 PMC5555839 28198106

[64] S. Gampala , F. Shah , X. Lu , H.‐r. Moon , O. Babb , N. U. Ganesh , G. Sandusky , E. Hulsey , L. Armstrong , A. L. Mosely , J. Experimental & Clinical Cancer Res. 2021, 40, 1. 10.1186/s13046-021-02046-x PMC8353735 34376225

[65] A. Kurynina , M. Erokhina , O. Makarevich , V. Y. Sysoeva , L. Lepekha , S. Kuznetsov , G. Onishchenko , Biochemistry 2018, 83, 200. 29625541 10.1134/S0006297918030021

[66] X. Huang , D. P. Cavalcante , H. E. Townley , J. Nanopart Res. 2020, 22, 23. 32435151 10.1007/s11051-019-4720-1 PMC7223038

[67] F. H. Osier , G. Feng , M. J. Boyle , C. Langer , J. Zhou , J. S. Richards , F. J. McCallum , L. Reiling , A. Jaworowski , R. F. Anders , K. Marsh , J. G. Beeson , BMC Med. 2014, 12, 108. 24980799 10.1186/1741-7015-12-108 PMC4098671

[68] R. Jungmann , M. S. Avendaño , J. B. Woehrstein , M. Dai , W. M. Shih , P. Yin , Nat. Methods 2014, 11, 313. 24487583 10.1038/nmeth.2835 PMC4153392

[69] J. Schnitzbauer , M. T. Strauss , T. Schlichthaerle , F. Schueder , R. Jungmann , Nat. Protoc. 2017, 12, 1198. 28518172 10.1038/nprot.2017.024

[70] E. Lerner , T. Cordes , A. Ingargiola , Y. Alhadid , S. Chung , X. Michalet , S. Weiss , Science 2018, 359, eaan1133. 29348210 10.1126/science.aan1133 PMC6200918

[71] H. Kobayashi , P. L. Choyke , Accounts of Chem. Res. 2011, 44, 83. 10.1021/ar1000633 PMC3040277 21062101

[72] H. Lee , A. K. R. Lytton‐Jean , Y. Chen , K. T. Love , A. I. Park , E. D. Karagiannis , A. Sehgal , W. Querbes , C. S. Zurenko , M. Jayaraman , C. G. Peng , K. Charisse , A. Borodovsky , M. Manoharan , J. S. Donahoe , J. Truelove , M. Nahrendorf , R. Langer , D. G. Anderson , Nat. Nanotechnol. 2012, 7, 389. 22659608 10.1038/nnano.2012.73 PMC3898745

[73] Y. Wang , I. Baars , I. Berzina , I. Rocamonde‐Lago , B. Shen , Y. Yang , M. Lolaico , J. Waldvogel , I. Smyrlaki , K. Zhu , R. A. Harris , B. Högberg , Nat. Nanotechnol. 2024. 10.1038/s41565-024-01676-4 PMC11405282 38951595

[74] S. M. Douglas , I. Bachelet , G. M. Church , Science 2012, 335, 831. 22344439 10.1126/science.1214081

[75] A. R. Chandrasekaran , Nat. Rev. Chem. 2021, 5, 225. 10.1038/s41570-021-00251-y PMC7873672 33585701

[76] S. Raniolo , S. Croce , R. P. Thomsen , A. H. Okholm , V. Unida , F. Iacovelli , A. Manetto , J. Kjems , A. Desideri , S. Biocca , Nanoscale 2019, 11, 10808. 31134260 10.1039/c9nr02006c

[77] M. M. C. Bastings , F. M. Anastassacos , N. Ponnuswamy , F. G. Leifer , G. Cuneo , C. Lin , D. E. Ingber , J. H. Ryu , W. M. Shih , Nano Lett. 2018, 18, 3557. 29756442 10.1021/acs.nanolett.8b00660

[78] S. Behzadi , V. Serpooshan , W. Tao , M. A. Hamaly , M. Y. Alkawareek , E. C. Dreaden , D. Brown , A. M. Alkilany , O. C. Farokhzad , M. Mahmoudi , Chem. Soc Rev. 2017, 46, 4218. 28585944 10.1039/c6cs00636a PMC5593313

[79] V. Jayashankar , A. L. Edinger , Nat. Commun. 2020, 11, 1121. 32111826 10.1038/s41467-020-14928-3 PMC7048872

[80] Y. Zhang , C. Commisso , Trends Cancer 2019, 5, 332. 31208695 10.1016/j.trecan.2019.04.002 PMC7325493

[81] F. Xiao , J. Li , K. Huang , X. Li , Y. Xiong , M. Wu , L. Wu , W. Kuang , S. Lv , L. Wu , X. Zhu , H. Guo , Am J. Cancer Res. 2021, 11, 14. 33520357 PMC7840718

[82] S. Song , Y. Zhang , T. Ding , N. Ji , H. Zhao , Front Oncol. 2020, 10, 570108. 33542897 10.3389/fonc.2020.570108 PMC7851083

[83] J. Cullis , D. Siolas , A. Avanzi , S. Barui , A. Maitra , D. Bar‐Sagi , Cancer Immunol. Res. 2017, 5, 182. 28108630 10.1158/2326-6066.CIR-16-0125 PMC5570452

[84] H. D. Moreau , C. Blanch‐Mercader , R. Attia , M. Maurin , Z. Alraies , D. Sanseau , O. Malbec , M.‐G. Delgado , P. Bousso , J.‐F. Joanny , Dev. Cell 2019, 49, e175. 10.1016/j.devcel.2019.03.024 30982662

[85] W. Yao , J. L. Rose , W. Wang , S. Seth , H. Jiang , A. Taguchi , J. Liu , L. Yan , A. Kapoor , P. Hou , Nature 2019, 568, 410. 30918400 10.1038/s41586-019-1062-1 PMC6661074

[86] M. Orth , P. Metzger , S. Gerum , J. Mayerle , G. Schneider , C. Belka , M. Schnurr , K. Lauber , Radiat. Oncol. 2019, 14, 141. 31395068 10.1186/s13014-019-1345-6 PMC6688256

[87] N. A. Ottenhof , R. F. de Wilde , A. Maitra , R. H. Hruban , G. J. Offerhaus , Patholog. Res. Int. 2011, 2011, 620601. 21512581 10.4061/2011/620601 PMC3068308

[88] H. Ying , P. Dey , W. Yao , A. C. Kimmelman , G. F. Draetta , A. Maitra , R. A. DePinho , Genes Dev. 2016, 30, 355. 26883357 10.1101/gad.275776.115 PMC4762423

[89] S. R. Choi , Y. Yang , K. Y. Huang , H. J. Kong , M. J. Flick , B. Han , Mater. Today Adv. 2020, 8, 100117. 34541484 10.1016/j.mtadv.2020.100117 PMC8448271

[90] A. M. Waters , C. J. Der , Cold Spring Harbor Perspectives in Med. 2018, 8, a031435. 10.1101/cshperspect.a031435 PMC5995645 29229669

[91] J. Bailey , A. Hendley , K. Lafaro , M. Pruski , N. Jones , J. Alsina , M. Younes , A. Maitra , F. McAllister , C. Iacobuzio‐Donahue , Oncogene 2016, 35, 4282. 26592447 10.1038/onc.2015.441

[92] J. Kleeff , M. Korc , M. Apte , C. La Vecchia , C. D. Johnson , A. V. Biankin , R. E. Neale , M. Tempero , D. A. Tuveson , R. H. Hruban , J. P. Neoptolemos , Nat. Rev. Disease Prim. 2016, 2, 16022. 27158978 10.1038/nrdp.2016.22

[93] C. Commisso , S. M. Davidson , R. G. Soydaner‐Azeloglu , S. J. Parker , J. J. Kamphorst , S. Hackett , E. Grabocka , M. Nofal , J. A. Drebin , C. B. Thompson , J. D. Rabinowitz , C. M. Metallo , M. G. Vander Heiden , D. Bar‐Sagi , Nature 2013, 497, 633. 23665962 10.1038/nature12138 PMC3810415

[94] H. R. Kim , S. J. Park , Y. S. Cho , M. K. Moyo , J. U. Choi , N. K. Lee , S. W. Chung , S. Kweon , J. Park , B. Kim , Y. G. Ko , J. H. Yeo , J. Lee , S. Y. Kim , Y. Byun , J. Control Release 2024, 372, 176. 38880331 10.1016/j.jconrel.2024.06.028

[95] F. Li , T. G. Cha , J. Pan , A. Ozcelikkale , B. Han , J. H. Choi , ChemBioChem 2016, 17, 1138. 27059426 10.1002/cbic.201600052 PMC5051347

[96] J. J. Rennick , A. P. Johnston , R. G. Parton , Nat. Nanotechnol. 2021, 16, 266. 33712737 10.1038/s41565-021-00858-8

[97] J. Rejman , V. Oberle , I. S. Zuhorn , D. Hoekstra , Biochemical J. 2004, 377, 159. 10.1042/BJ20031253 PMC1223843 14505488

[98] D. P. Logsdon , M. Grimard , M. Luo , S. Shahda , Y. Jiang , Y. Tong , Z. Yu , N. Zyromski , E. Schipani , F. Carta , Mol. Cancer Therapeutics 2016, 15, 2722. 10.1158/1535-7163.MCT-16-0253 PMC5097013 27535970

[99] S. Jones , X. Zhang , D. W. Parsons , J. C.‐H. Lin , R. J. Leary , P. Angenendt , P. Mankoo , H. Carter , H. Kamiyama , A. Jimeno , Science 2008, 321, 1801. 18772397 10.1126/science.1164368 PMC2848990

[100] H. Wiig , M. A. Swartz , Physiol. Rev. 2012, 92, 1005. 22811424 10.1152/physrev.00037.2011

[101] J. Varennes , H.‐r. Moon , S. Saha , A. Mugler , B. Han , PLoS Comput. Biol. 2019, 15, e1006961. 30970018 10.1371/journal.pcbi.1006961 PMC6476516

[102] N. S. H. Motlagh , P. Parvin , F. Ghasemi , F. Atyabi , Biomed. Optics Express 2016, 7, 2400. 10.1364/BOE.7.002400 PMC4918592 27375954

