# Supplementary material for: DNA Origami‐Cyanine Nanocomplex for Precision Imaging of KRAS‐Mutant Pancreatic Cancer Cells
Source: Adv Sci (Weinh). 2025 Feb 14;12(19):2410278. doi: 10.1002/advs.202410278 (PMC12097083; doi:10.1002/advs.202410278)
Supplement: Supplementary file 1 — Supporting Information [file ADVS-12-2410278-s001.docx]

## Supporting Information

DNA Origami-Cy5 Nanocomplex for Precision Imaging of KRAS-mutant Pancreatic Cancer Cells

Hye-ran Moon, Yancheng Du, Sae Rome Choi, Seongmin Seo, Cih Cheng, Bennett D. Elzey, Jong Hyun Choi*, Bumsoo Han *

**Quantitative assessment of the tumor-targeting efficacy of the DO-Cy5 nanocomplex**

The quantitative assessment of the tumor-targeting efficacy of Do-Cy nanocomplex is determined by measuring temporal drug accumulation in each cell. The measurement of the drug accumulation was assessed separately for each cell type. To maintain accuracy and prevent misclassification between cell types, regions corresponding to CAFs were explicitly excluded from the analysis of PCC regions. PCC areas were identified based on their distinct morphological characteristics.


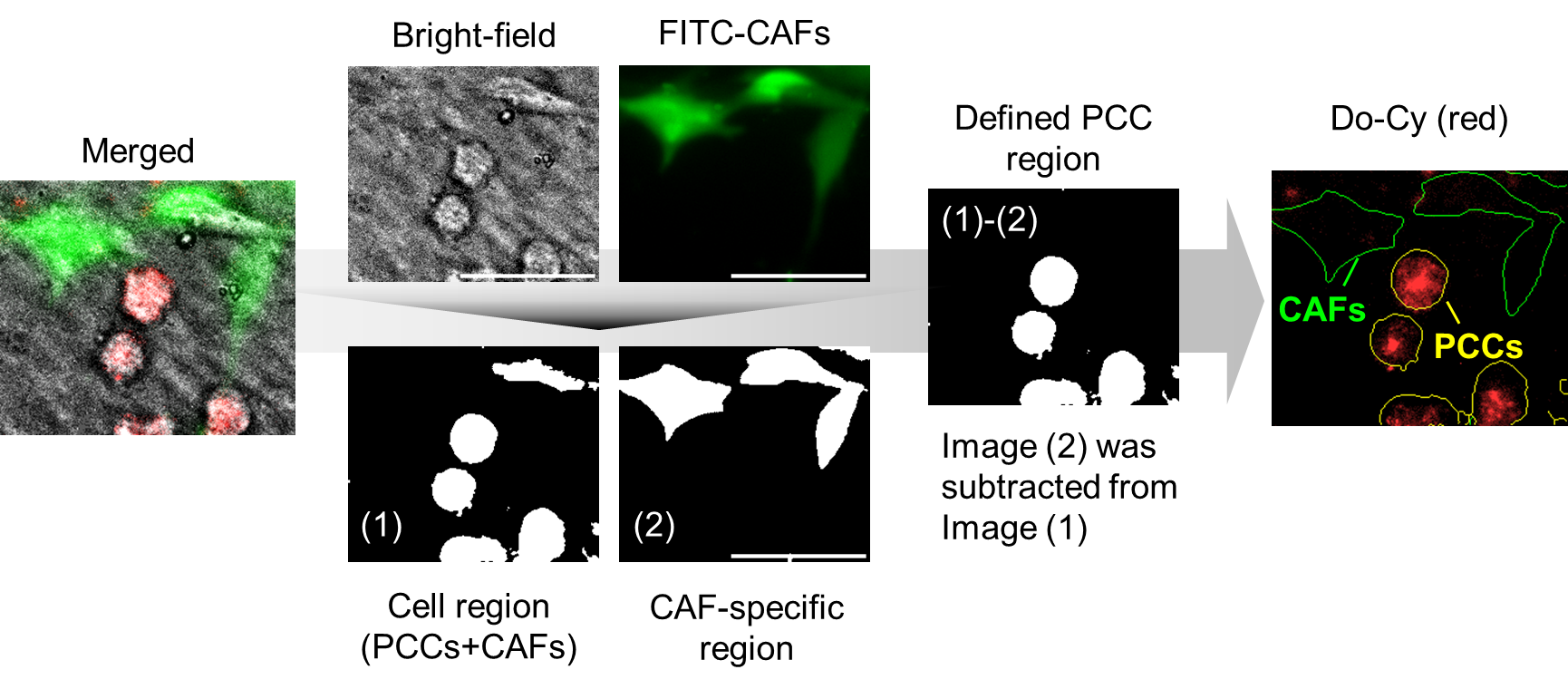


Following the quantitative measurement of the accumulation patterns in the dynamic transport system using the 3D PDAC MPS model (**Figure 6**), we further analyzed the intracellular transport of DO-Cy5 using the simplified binding kinetics model with mass conservation introduced in our previous studies:

$$\frac{d}{dt}(C_{cell}\cdot V_{cell})=J_{in}-J_{out}$$

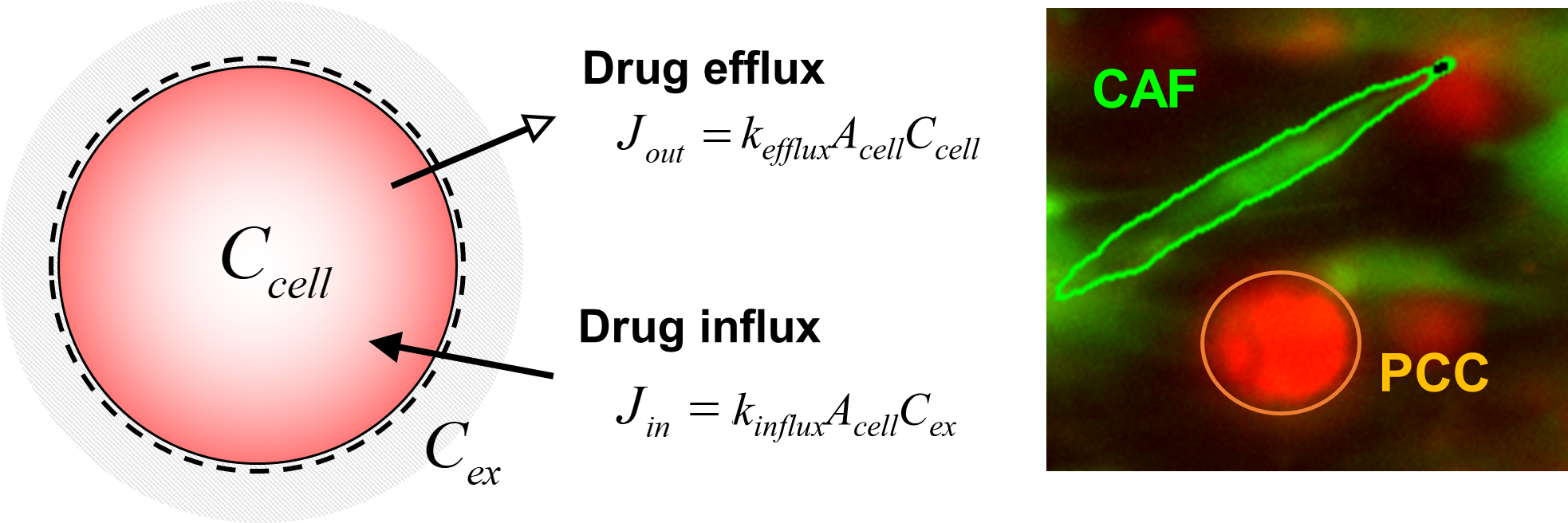


where *C_cell_* is an intracellular drug concentration in the cellular cytosol and *V_cell_* is an estimated volume of each cell. In the model, transient drug accumulation is regulated by an equilibrium between drug influx (*J_in_*) and efflux (*J_out_*) at the cell membrane. The cell's drug uptake affinity (*k_influx_*) at the cell surface and drug concentration at the extracellular region near the cells (*C_ex_*) govern the drug influx, while the drug efflux depends on the drug dissociation affinity (*k_efflux_*) and intracellular drug concentration (*C_cell_*). We used the measurement data to determine *k_influx_* and *k_efflux_* for nonlinear curve fitting in MATLAB, assuming constant parameters. This approach allowed us to quantitatively assess the cellular capacity for drug uptake and efflux.

**
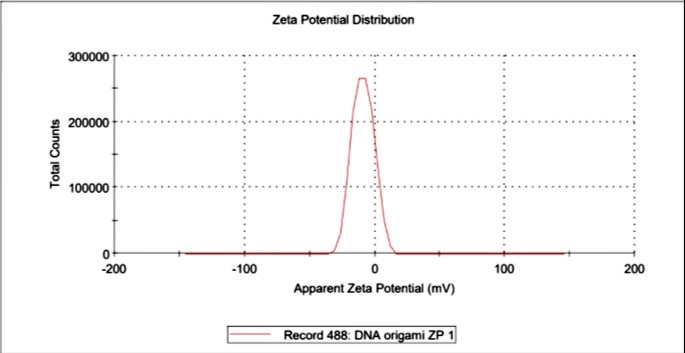
**

**Figure S1. Zeta potential distribution of DNA Origami-Cy5**

**
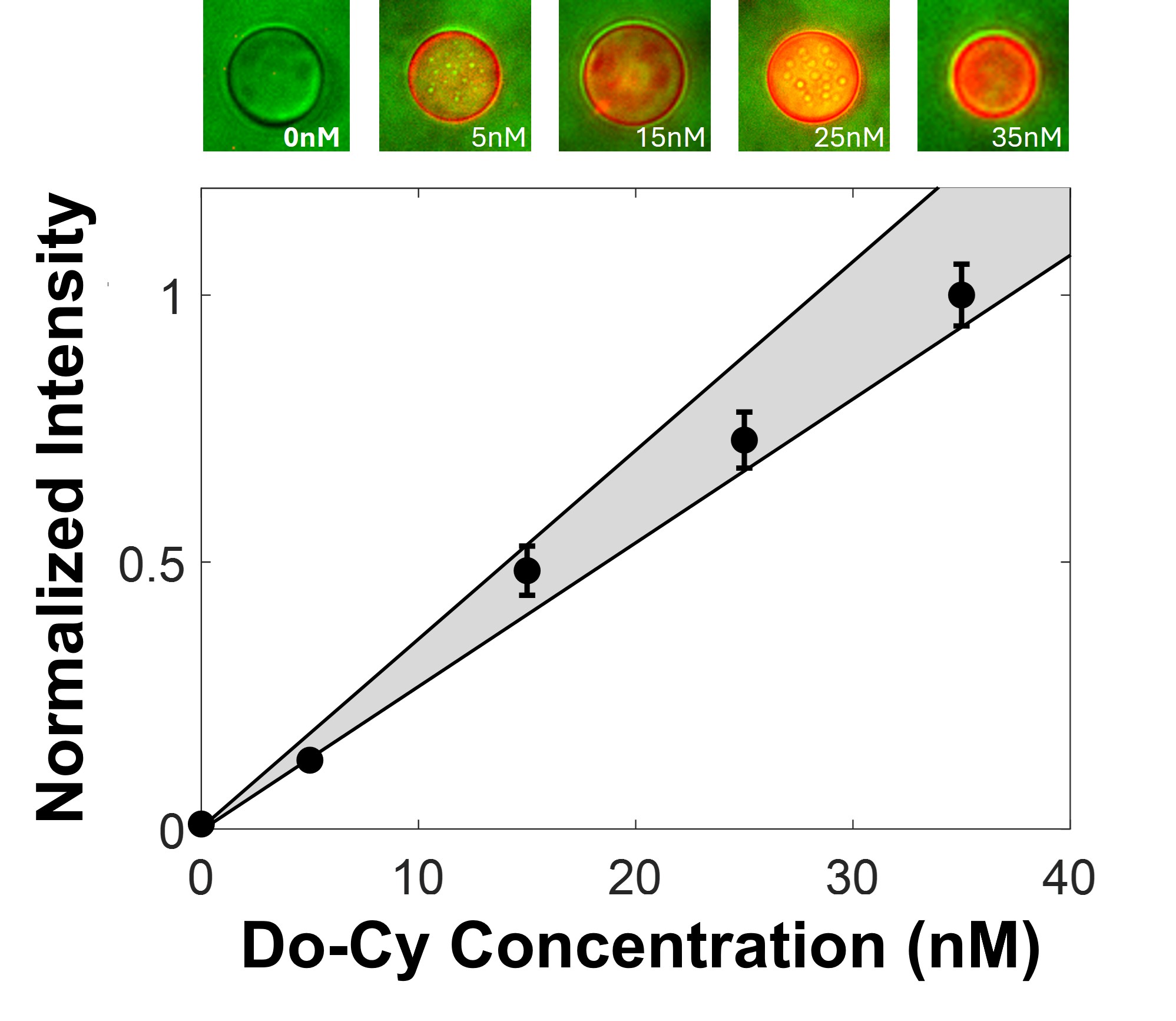
**

**Figure S2. Calibration of the Do-Cy concentration with fluorescence intensity.** Do-Cy samples at concentrations of 0, 5, 15, 25, and 35 nM in giant unilamellar vesicles (GUVs) were analyzed by fluorescence imaging. Ten measurements were conducted for each concentration, and the average fluorescence intensity values were plotted. The results provide a reliable dataset for calibrating Do-Cy concentration, enabling the quantitative assessment of DNA origami uptake levels in PCCs based on fluorescence intensity. Details of GUV synthesis and sample preparation were reported elsewhere.[1]

**
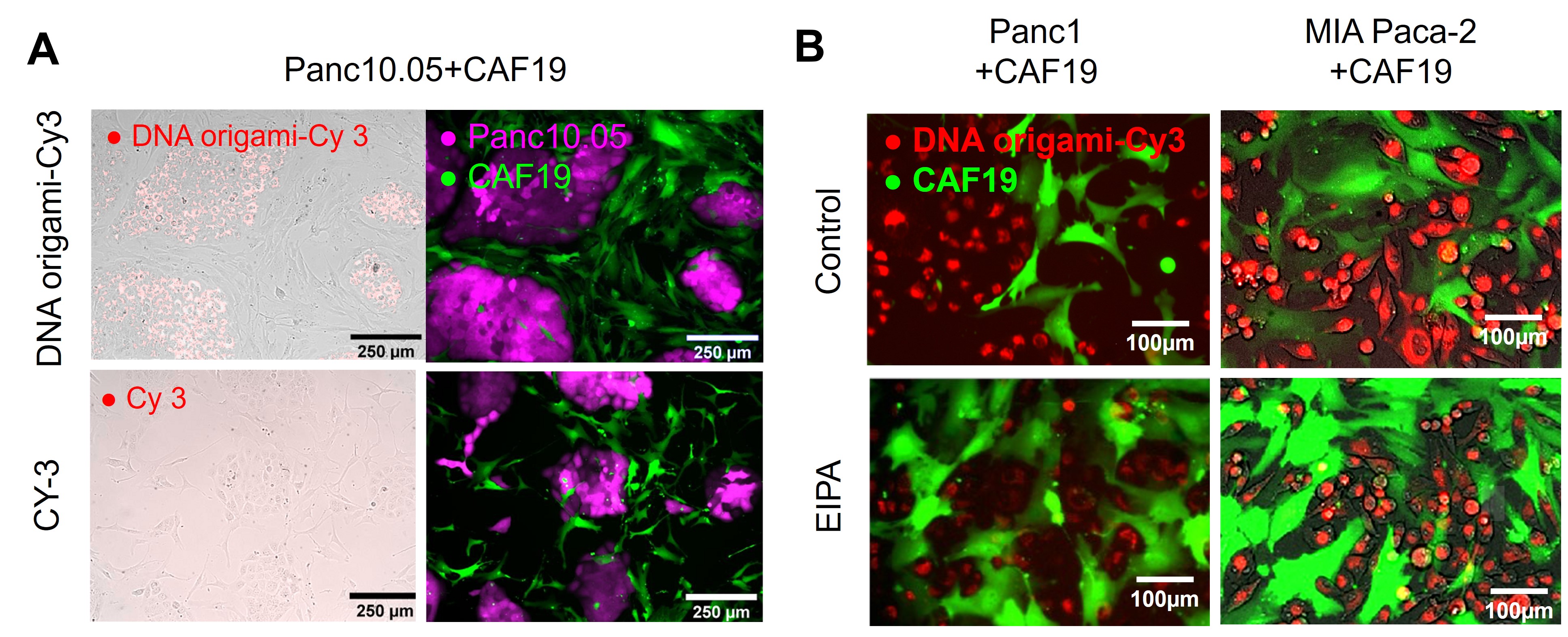
Figure S3. DNA origami – Cy3 nanocomplex accumulation in pancreatic tumor cells preferentially more than stroma cells** (A) Fluorescence micrograph of DNA origami-Cy3 and Cy3 (control) accumulation after 24-hour exposure in 2D monolayer of pancreatic cancer cells (Panc10.05) and CAFs (CAF19). Magenta indicates the DNA-origami Cy3 nanocomplex and Cy3 accumulated in the cells, red indicates Panc10.05 cells, and green indicates CAF19 cells. (B) DNA origami-Cy3 accumulation in pancreatic cancer cells with and without EIPA, a macropinocytosis inhibitor. Red represents DNA origami-Cy3 nanocomplex.

**
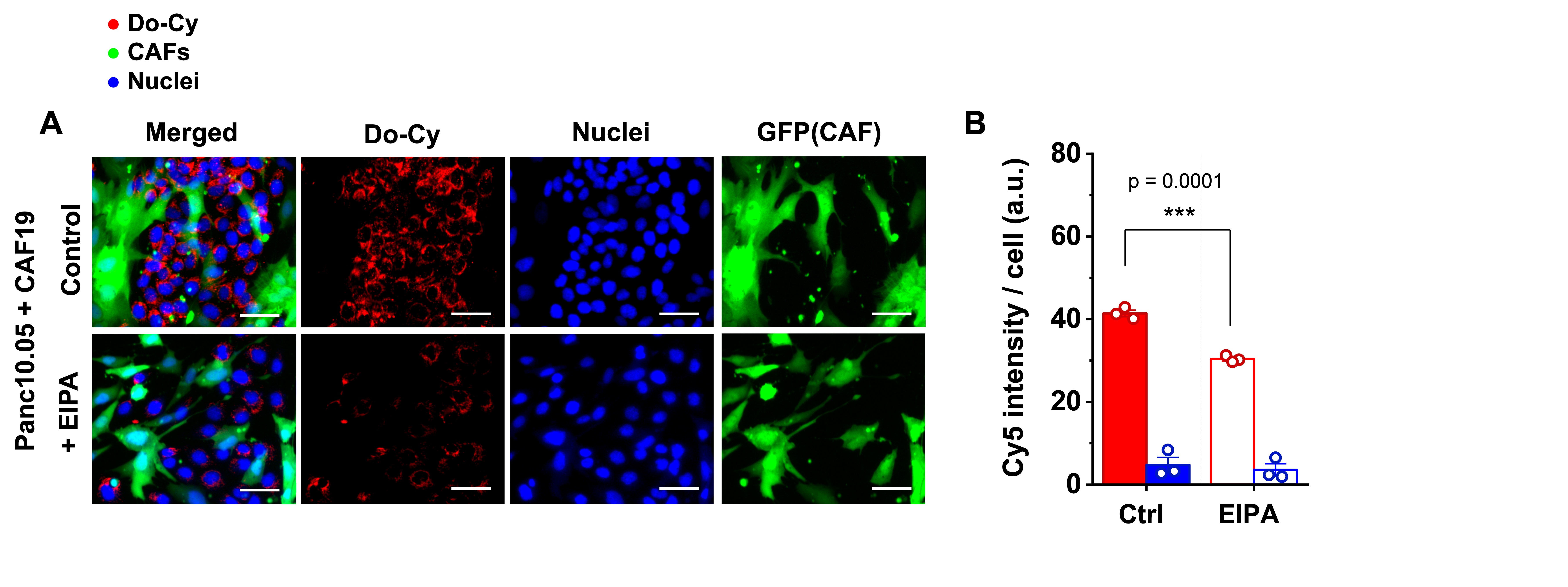
**

**Figure S4.** (A) Do-Cy accumulation with and without EIPA, a macropinocytosis inhibitor. (B) Relative Cy intensity accumulated per cells. Red represents Do-Cy, nuclei are blue, and GFP-transfected CAFs are green. Bars indicate Mean ± S.E., and dots indicate each data point (n = 3). P-values of <0.05, <0.01, and <0.001 are represented as *, **, and *** respectively (Student t-test). Scale bars indicate 100 µm.

**
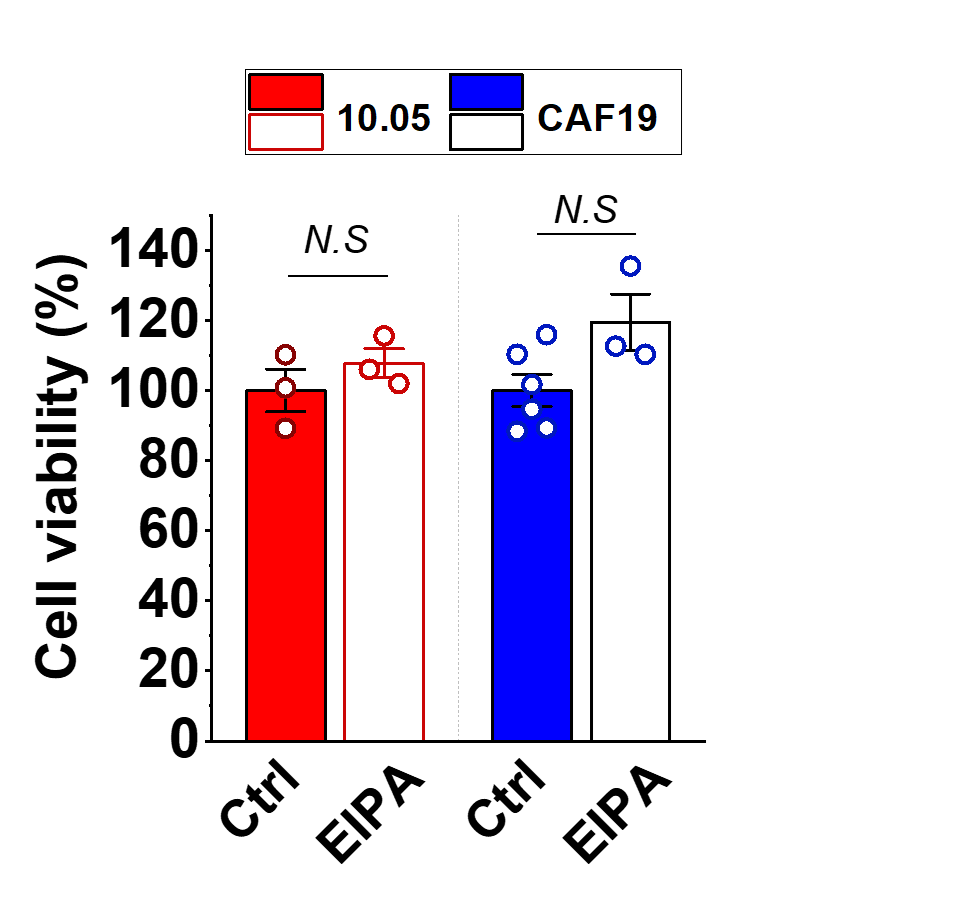
**

**Figure S5. MTS cell viability assay for 10.05 and CAF19 with and without 50µM of EIPA treatment.**

**
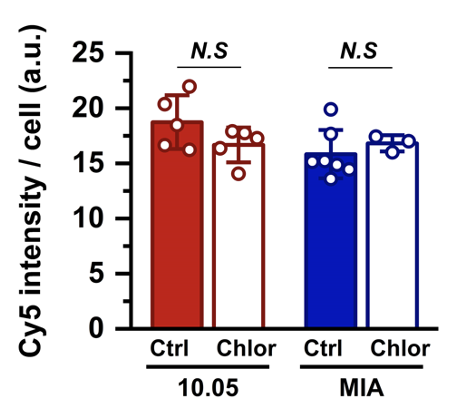
**

**Figure S6. Investigation of inhibition of endocytosis mechanism for Do-Cy.** Chlorpromazine prevents clathrin-mediated endocytosis by inhibiting cell surface clathrin lattice organization.[2] Bars indicate Mean ± S.E., and dots indicate each data point (n ≥ 5). P-values of <0.05, <0.01, and <0.001 are represented as *, **, and *** respectively. *N.S* indicates no statistical significance with p-values ≥ 0.05.

**
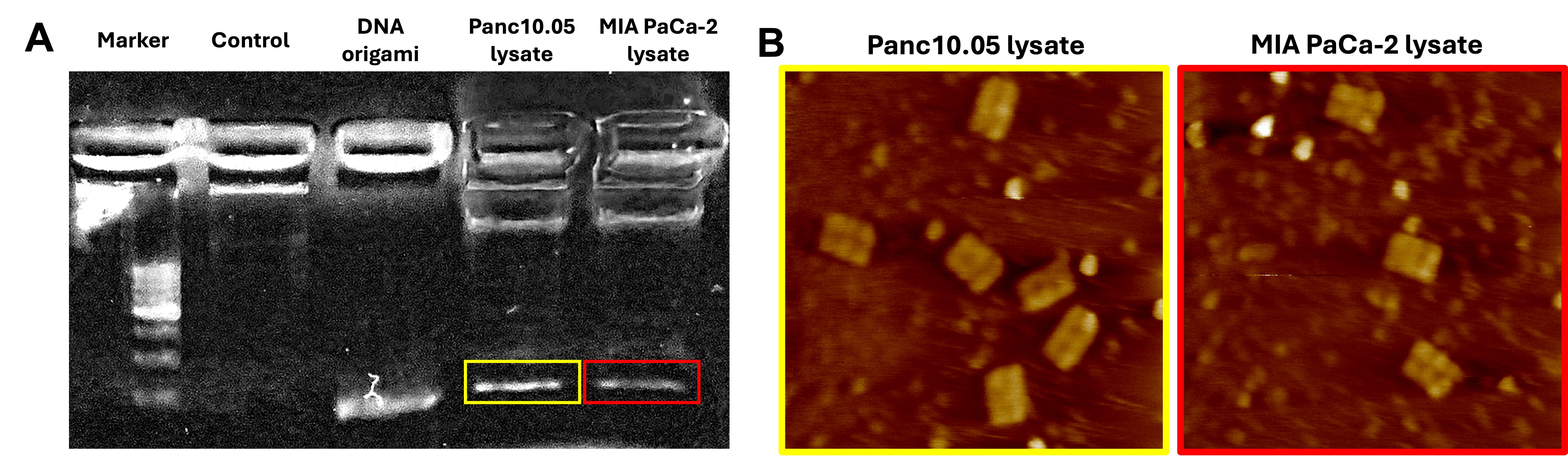
**

**Figure S7. Stability Test of Do-Cy in PCC Lysates.** (A) Do-Cy samples were incubated with PCC lysates for at least 16 hours, and their stability was evaluated using agarose gel electrophoresis. From left to right: 1 kb DNA ladder (brightest band at 5 kb), control (cell lysate without Do-Cy), as-prepared DNA origami, Do-Cy incubated in lysates from Panc10.05, and MIA PaCa-2 cells. The slightly slower migration of DNA origami bands in PCC lysates compared to the as-prepared sample is due to interactions with cellular components. (B) AFM images of the PCC lysates show DNA origami tubules resembling those in the as-prepared DNA origami shown in Figure 1C, demonstrating the biostability of Do-Cy in cell lysates.

**
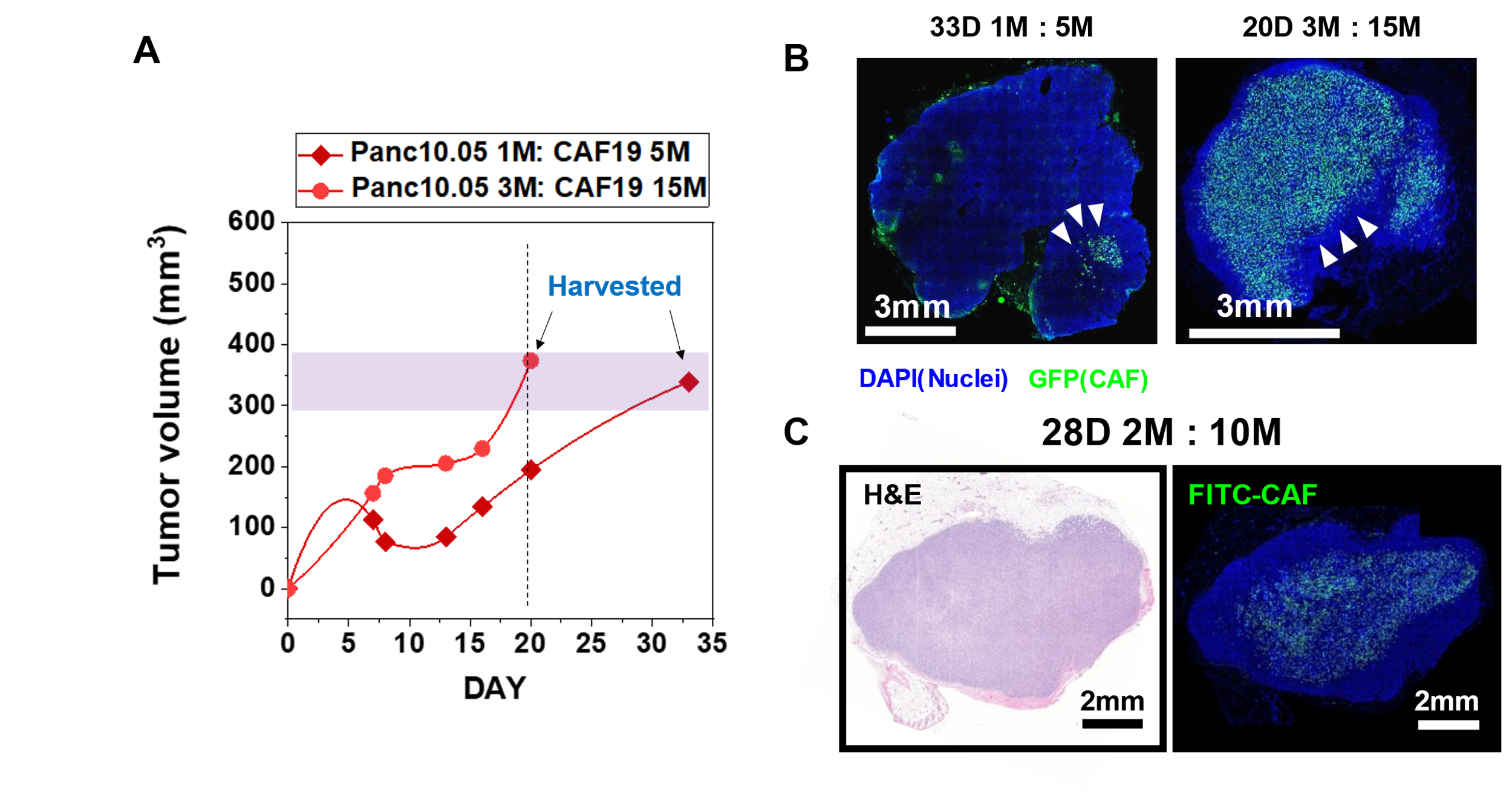
**

**Figure S8. Optimization in developing xenograft mouse models inoculating PCCs with CAFs.** (A) The effect of inoculation cell density in tumor size growth. Tumors were harvested when their volume reached the range of 300-400 mm² - on day 33 for 1X10^6^ PCC : 5X10^6^ CAF (33D 1M:5M), and on day 20 for 3X10^6^ PCC : 15X10^6^ CAF (33D 3M:15M). (B) Immunohistochemistry to determine the population of GFP-transfected CAFs. The CAF population appeared diminished for the 33D 1M:5M condition, whereas for 20D 3M:15M, CAFs were still predominant over PCCs. As the tumors grew beyond 30 days, the CAF population decreased. To ensure an adequate CAF population, we optimized the inoculation density to 2X10^6^ PCC : 10X10^6^ CAF and expected to be ready for DNA-origami injection by DAY28. (C) Histological staining with hematoxylin and eosin (H&E) and immunohistochemistry for nuclei (blue) and FITC (green) confirmed the presence of CAFs in tumors of optimal size at 28 days for the inoculation cell density of 2M:10M.

**
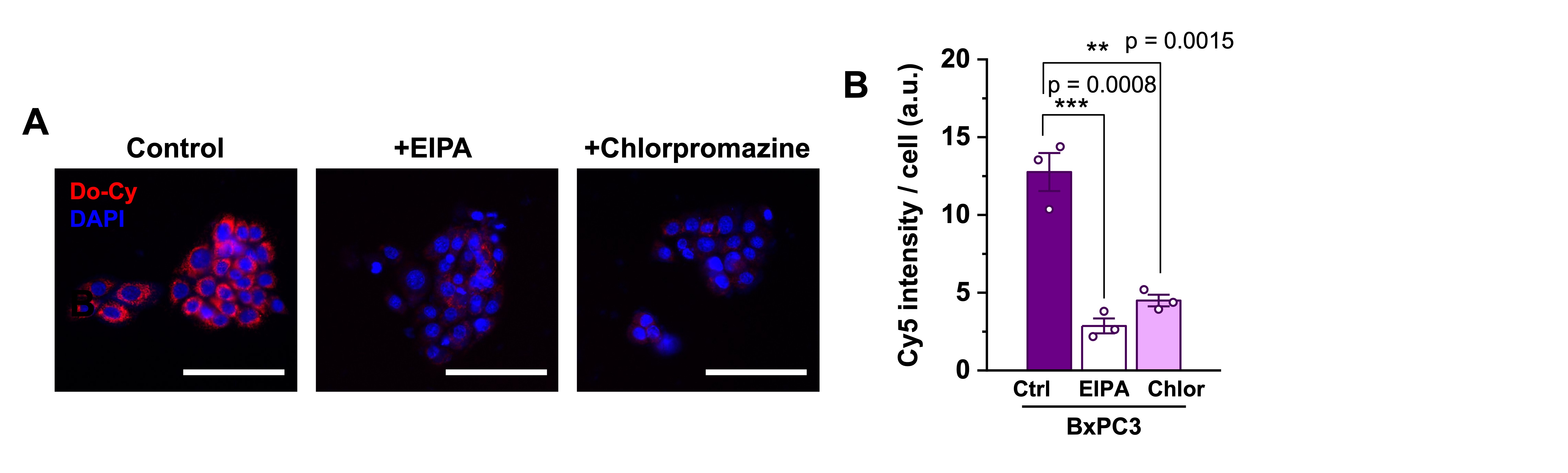
**

**Figure S9. Do-Cy accumulation in BxPC3.** (A) BxPC3 cells, reported to have no KRAS mutation, demonstrates noticeable accumulation of Do-Cy. Inhibition of both macropinocytosis with EIPA and clathrin-mediated endocytosis with Chlorpromazine significantly reduces Do-Cy uptake, suggesting that experiments investigating Do-Cy uptake mechanism for non-KRAS mutant PCCs warrant investigation. Red represents Do-Cy and nuclei are blue. Scale bar – 100 µm. (B) Relative Cy intensity accumulated per cell. Bars indicate Mean ± S.E., dots indicate each data point (n = 3).

**Table S1**

Cy5 modified sequences.

| Name | Sequence |
| --- | --- |
| Cy5-13 | /5Cy5/GC GGT TCA GA |
| Cy5-16 | /5Cy5/AT CCT GGA TG |

**Table S2** 70 nm Tube Origami Staples

Sequences with red color are used to bind Cy5 fluorophores.

| sTop[03, 05] | GTAATATCCGCCAGAATCCTGAGAGTATAACG |
| --- | --- |
| sTop[03, 09] | GAGTAGAAGTGAGGCCACCGAGTAGAGCGGGC |
| sTop[03, 17] | AAAATCCCTGAGTGTTGTTCCAGTCGATTTAG |
| sTop[03, 21] | AAATCCTGCTATTAAAGAACGTGGAAGCACTA |
| sTop[03, 25] | AGCGGTCCGCGAAAAACCGTCTATCAAATCAA |
| sTop[05, 05] | AGTCTTTACGCTCAATCGTCTGAACCTTGCTG |
| sTop[05, 09] | CGTGGCACGGCAGATTCACCAGTCACTTGCCT |
| sTop[05, 17] | TCCAGTCGCGGCCAACGCGCGGGGAAATCGGC |
| sTop[05, 21] | CACATTAATTGGGCGCCAGGGTGGGCAGGCGA |
| sTop[05, 25] | AAAGCCTGTGAGACGGGCAACAGCTTGCAGCA |
| sTop[07, 05] | AAAGGAATTAAAACAGAGGTGAGGTGGCTATT |
| sTop[07, 09] | CAATATCTCGCCTGCAACAGTGCCTAAGAATA |
| sTop[07, 17] | AAAACGACACTCTAGAGGATCCCCGCCCGCTT |
| sTop[07, 21] | TAACGCCATTCGTAATCATGGTCAAGCTAACT |
| sTop[07, 25] | AGGGGGATGAAATTGTTATCCGCTTAAAGTGT |
| sTop[09, 05] | TCATATTCATAATACATTTGAGGAAACAGTTG |
| sTop[09, 09] | CAAAGAAATTTACAAACAATTCGACCCTCAAT |
| sTop[09, 17] | CGACGACAGCTTTCCGGCACCGCTGACGTTGT |
| sTop[09, 21] | ATCGTAACAGGCAAAGCGCCATTCAAGTTGGG |
| sTop[09, 25] | ATGGGATAAACTGTTGGGAAGGGCCTGGCGAA |
| sTop[11, 05] | TGAATACCATGGAAGGGTTAGAACAATTATCA |
| sTop[11, 09] | GGAGAAACATTTGCACGTAAAACATTGCGGAA |
| sTop[11, 17] | CATTAAATGGAACGCCATCAAAAATGAGGGGA |
| sTop[11, 21] | AATTGTAATTCCTGTAGCCAGCTTATGGGCGC |
| sTop[11, 25] | AAAAACAGTGAGCGAGTAACAACCTGACCGTA |
| sTop[13, 05] | AAAACATAAACAAACATCAAGAAAGATTGCTT |
| sTop[13, 09] | CGCTATTATTAACAATTTCATTTGTTACATCG |
| sTop[13, 17] | TGATAAATTCTACAAAGGCTATCAAAAATTCG |
| sTop[13, 21] | TCACCATCTCTGGAGCAAACAAGAAATATTTA |
| sTop[13, 25] | CAAAAGGGATCGTAAAACTAGCATAAAGCCCC |
| sTop[15, 05] | ATACCGACGAGACTACCTTTTTAAAATCCTTG |
| sTop[15, 09] | TTCATCTTGGTTATATAACTATATTAAATCGT |
| sTop[15, 17] | AATAAAGCAAACATTATGACCCTGGTTCTAGC |
| sTop[15, 21] | ACAGGCAAGAAGCCTTTATTTCAACAGTCAAA |
| sTop[15, 25] | AATAGTAGTTTAGAACCCTCATATTAAAGATT |
| sTop[17, 05] | CAAAAGGTTTTAGTATCATATGCGGGTTTGAA |
| sTop[17, 09] | CGAGCCAGCAGTATAAAGCCAACGTAGTTAAT |
| sTop[17, 17] | TACGGTGTCCAATTCTGCGAACGAAATTAAGC |
| sTop[17, 21] | TAGCTCAACATTAGATACATTTCGTAAATCAT |
| sTop[17, 25] | GGCTTAGATGTTTAGCTATATTTTATTCTACT |
| sTop[19, 05] | TTTTTATTTATCAACAATAGATAAAGTACCGA |
| sTop[19, 09] | AAGTACCGATAATATCCCATCCTAGGCATTTT |
| sTop[19, 17] | GCGGATTGTTCAAATATCGCGTTTAACTAAAG |
| sTop[19, 21] | TCTTTACCGCGAACCAGACCGGAATAATGCTG |
| sTop[19, 25] | AAAACGAGCAGGATTAGAGAGTACTTGCGGAT |
| sTop[21, 05] | AGTTACAAATTCTAAGAACGCGAGGCAAGCCG |
| sTop[21, 09] | CTAACGAGCCCGACTTGCGGGAGGATTAAACC |
| sTop[21, 17] | TTTACCAGTTGCAAAAGAAGTTTTGAAGCAAA |
| sTop[21, 21] | GCATAGTAGTAAAATGTTTAGACTAAATCAGG |
| sTop[21, 25] | TGCAGATATGCGGAATCGTCATAACAGTTCAG |
| sTop[23, 05] | AGAGCAAGTGAAAATAGCAGCCTTAATTTGCC |
| sTop[23, 09] | AACCCACATAAAAACAGGGAAGCGTACCAACG |
| sTop[23, 17] | ATCATTGTATTATACCAGTCAGGAAACCCTCG |
| sTop[23, 21] | ATTGGGCTCTACGTTAATAAAACGATTACGAG |
| sTop[23, 25] | GCCCTGACTTATTACAGGTAGAAATCAACTAA |
| sTop[25, 05] | CCACGGAAAACAAAGTTACCAGAACAATAATA |
| sTop[25, 09] | AGGTGGCACAATAATAACGGAATAAGAGAGAT |
| sTop[25, 17] | TAAGGGAAAACGGTGTACAGACCACAACTTTA |
| sTop[25, 21] | CTTAGCCGGACCTTCATCAAGAGTAGTAGTAA |
| sTop[25, 25] | TTGTGTCGGGATATTCATTACCCATAAGGCTT |
| sTop[27, 05] | ACCAATGACGACATTCAACCGATTCAAAGACA |
| sTop[27, 09] | GCACCATTATATTGACGGAAATTAATACATAA |
| sTop[27, 17] | AGTTTCCAAGGCACCAACCTAAAAGTCAATCA |
| sTop[27, 21] | GGCTTTGAATACACTAAAACACTCCCATGTTA |
| sTop[27, 25] | AGCATCGGGATTATACCAAGCGCGCTGATAAA |
| sTop[29, 05] | CAGAGCCGTAGCGCGTTTTCATCGGAAACGTC |
| sTop[29, 09] | CTCAGAACCCCCCTTATTAGCGTTCACCAGTA |
| sTop[29, 17] | GCTTGCTTATAGTTGCGCCGACAACATGAGGA |
| sTop[29, 21] | CAAAAGGACCACGCATAACCGATAGCTACAGA |
| sTop[29, 25] | TTTCACGTCTTGCAGGGAGTTAAACGAAAGAC |
| sTop[31, 05] | ACAGTGCCGGCCTTGATATTCACAACCACCCT |
| sTop[31, 09] | AATAAGTTTTAAAGCCAGAATGGACCGCCACC |
| sTop[31, 17] | ACAGACAGGTCGTCTTTCCAGACGGTTTATCA |
| sTop[31, 21] | ACCAGTACCTGTATGGGATTTTGCAAAGGCTC |
| sTop[31, 25] | GGAACCCAGTTTCAGCGGAGTGAGAATAATTT |
| sBot[02, 04] | GAACGGTACAGAACAATATTACCGAATACCTA |
| sBot[02, 08] | TATAATCAGAACTCAAACTATCGGATGGATTA |
| sBot[02, 12] | TGTCCATCGATTAGTAATAACATCACACGACC |
| sBot[02, 20] | AGAGTCCATTTGATGGTGGTTCCGAGAGGCGG |
| sBot[02, 24] | GTCAAAGGACGCTGGTTTGCCCCATTTTTCTT |
| sBot[04, 04] | CATTTTGAATGCGCGAACTGATAGAACCACCA |
| sBot[04, 08] | TTTACATTAGACAATATTTTTGAACGGTCAGT |
| sBot[04, 12] | AGTAATAATTCTGACCTGAAAGCGACGCTGAG |
| sBot[04, 20] | TTTGCGTATTGCGTTGCGCTCACTGGGTACCG |
| sBot[04, 24] | TTCACCAGGGGTGCCTAATGAGTGTAGCTGTT |
| sBot[06, 04] | GCAGAAGATGAGGAAGGTTATCTATTAGAGCC |
| sBot[06, 08] | ATTAACACGGTCAGTTGGCAAATCTTTAGAAG |
| sBot[06, 12] | AGCCAGCAAACCTCAAATATCAAACAACTCGT |
| sBot[06, 20] | AGCTCGAAGGGTTTTCCCAGTCACTCTGGTGC |
| sBot[06, 24] | TCCTGTGTGTGCTGCAAGGCGATTGCCATTCA |
| sBot[08, 04] | GTCAATAGCTGATTATCAGATGATATTATACT |
| sBot[08, 08] | TATTAGACCCACCAGAAGGAGCGGCTACCATA |
| sBot[08, 12] | ATTAAATCGAGTAACATTATCATTGAAATAAA |
| sBot[08, 20] | CGGAAACCCGTGCATCTGCCAGTTTAATTCGC |
| sBot[08, 24] | GGCTGCGCGGTCACGTTGGTGTAGTCATCAAC |
| sBot[10, 04] | TCTGAATAAAGTTACAAAATCGCGCAAAAGAA |
| sBot[10, 08] | TCAAAATTAATAACGGATTCGCCTACAAAATT |
| sBot[10, 12] | GAAATTGCACAGTAACAGTACCTTAATTACCT |
| sBot[10, 20] | GTCTGGCCACGTTAATATTTTGTTGGTCATTG |
| sBot[10, 24] | ATTAAATGGAAGATTGTATAAGCAGAATCGAT |
| sBot[12, 04] | GATGATGAGCGATAGCTTAGATTAAAAATCAT |
| sBot[12, 08] | AATTACATATTAATTTTCCCTTAGCCTCCGGC |
| sBot[12, 12] | TTTTTAATAATAACCTTGCTTCTGGTAAATGC |
| sBot[12, 20] | CCTGAGAGAATATGATATTCAACCTAATACTT |
| sBot[12, 24] | GAACGGTATGAGAAAGGCCGGAGACGCAAGGA |
| sBot[14, 04] | AGGTCTGACGTGTGATAAATAAGGTACTAGAA |
| sBot[14, 08] | TTAGGTTGCTGACCTAAATTTAATTTATACAA |
| sBot[14, 12] | TGATGCAATTTTTCAAATATATTTCTCAACAG |
| sBot[14, 20] | TTGCGGGAGGCAAAGAATTAGCAAGTAGATTT |
| sBot[14, 24] | TAAAAATTTAGCATTAACATCCAACAAATGGT |
| sBot[16, 04] | AAAGCCTGAAAGTAATTCTGTCCAGCAGAACG |
| sBot[16, 08] | ATTCTTACTAATAAGAGAATATAAGTCCTGAA |
| sBot[16, 12] | TAGGGCTTATGTAATTTAGGCAGAATTTACGA |
| sBot[16, 20] | AGTTTGACCATGTTTTAAATATGCTAATTCGA |
| sBot[16, 24] | CAATAACCGCTTAATTGCTGAATAGCAAACTC |
| sBot[18, 04] | CGCCTGTTTTCATCGTAGGAATCAAGAAGGCT |
| sBot[18, 08] | CAAGAAAACACTCATCGAGAACAAGCGTTTTA |
| sBot[18, 12] | GCATGTAGCATTCCAAGAACGGGTTTTTGAAG |
| sBot[18, 20] | GCTTCAAACTGACTATTATAGTCAGCCAGAGG |
| sBot[18, 24] | CAACAGGTAATGACCATAAATCAAGGATAGCG |
| sBot[20, 04] | TATCCGGTAATAAACAGCCATATTTGTTTAAC |
| sBot[20, 08] | GCGAACCTCGTCTTTCCAGAGCCTTACAGAGA |
| sBot[20, 12] | CCTTAAATATTTTATCCTGAATCTCATTAGAC |
| sBot[20, 20] | GGGTAATAAGAGCAACACTATCATCGTTGGGA |
| sBot[20, 24] | TCCAATACCATAACGCCAAAAGGAAACTAACG |
| sBot[22, 04] | GTCAAAAAAAACAATGAAATAGCAAGTAAGCA |
| sBot[22, 08] | GAATAACAAGAATTGAGTTAAGCCGGAAACCG |
| sBot[22, 12] | GGGAGAATATTGAGCGCTAATATCCCCAAAAG |
| sBot[22, 20] | AGAAAAATTGAGATGGTTTAATTTGGCGCATA |
| sBot[22, 24] | GAACAACAGAGAAACACCAGAACGAATCTTGA |
| sBot[24, 04] | GATAGCCGTAAGTTTATTTTGTCAGCCAAAGA |
| sBot[24, 08] | AGGAAACGACATATAAAAGAAACGGAGGGAGG |
| sBot[24, 12] | AACTGGCACAAACGTAGAAAATACTTCATTAA |
| sBot[24, 20] | GGCTGGCTGAACGAGGCGCAGACGCGAAAGAG |
| sBot[24, 24] | CAAGAACCAAATCCGCGACCTGCTATCTTTGA |
| sBot[26, 04] | CAAAAGGGAACCATCGATAGCAGCCTTTAGCG |
| sBot[26, 08] | GAAGGTAAACCATTAGCAAGGCCGGCATTTTC |
| sBot[26, 12] | AGGTGAATTTAGAGCCAGCAAAATTGCCATCT |
| sBot[26, 20] | GCAAAAGAGGACTAAAGACTTTTTTGACAACA |
| sBot[26, 24] | CCCCCAGCAACGAGGGTAGCAACGTATTCGGT |
| sBot[28, 04] | TCAGACTGCCACCAGAACCACCACGGCAGGTC |
| sBot[28, 08] | GGTCATAGCGCCACCCTCAGAGCCAACAAATA |
| sBot[28, 12] | TTTCATAAACCGCCTCCCTCAGAGAAGCGCAG |
| sBot[28, 20] | ACCATCGCGCCTTTAATTGTATCGTTAGTAAA |
| sBot[28, 24] | CGCTGAGGTGAAAATCTCCAAAAATAAACAAC |
| sBot[30, 04] | AGACGATTCGTATAAACAGTTAATAAACATGA |
| sBot[30, 08] | AATCCTCATTAACGGGGTCAGTGCCAAGAGAA |
| sBot[30, 12] | TCTCTGAATTGATGATACAGGAGTTCAGTACC |
| sBot[30, 20] | TGAATTTTAAACTACAACGCCTGTCACCGTAC |
| sBot[30, 24] | TTTCAACATGTACCGTAACACTGATCAGAACC |
| seam[02, 16] | GATAGGGTTTATAAATCAAAAGAAGTAGCAATCATCCAGGAT |
| seam[03, 13] | ACTTCTTTACGCAAATTAACCGTTTAGCCCGATCTGAACCGC |
| seam[04, 16] | TAATGAATGGAAACCTGTCGTGCCAACAGAGACATCCAGGAT |
| seam[05, 13] | TAGAACCCAAGGGACATTCTGGCCAGCTGCATTCTGAACCGC |
| seam[06, 16] | GCAGGTCGGGCCAGTGCCAAGCTTAAGCATCACATCCAGGAT |
| seam[07, 13] | CCTTGCTGGCAAATGAAAAATCTAGCATGCCTTCTGAACCGC |
| seam[08, 16] | TCCAGCCAGTATCGGCCTCAGGAATTAATTTTCATCCAGGAT |
| seam[09, 13] | AAAAGTTTCTTTGCCCGAACGTTAGATCGCACTCTGAACCGC |
| seam[10, 16] | AACCAATATTTTGTTAAATCAGCTAACGTCAGCATCCAGGAT |
| seam[11, 13] | ATGAATATGTAGATTTTCAGGTTTCATTTTTTTCTGAACCGC |
| seam[12, 16] | TTGAGAGATAATGCCGGAGAGGGTTCAATATACATCCAGGAT |
| seam[13, 13] | TGTGAGTGGGAAACAGTACATAAAAGCTATTTTCTGAACCGC |
| seam[14, 16] | TGTACCAACTCAGAGCATAAAGCTAAGAACGCCATCCAGGAT |
| seam[15, 13] | GAGAAAACATCCAATCGCAAGACAAAATCGGTTCTGAACCGC |
| seam[16, 16] | GTTGATTCCTGGAAGTTTCATTCCATTTAACACATCCAGGAT |
| seam[17, 13] | ACGCCAACAATTGAGAATCGCCATATATAACATCTGAACCGC |
| seam[18, 16] | CGAAAGACCATCAAAAAGATTAAGGGCTGTCTCATCCAGGAT |
| seam[19, 13] | TTCCTTATAAACCAATCAATAATCAGGAAGCCTCTGAACCGC |
| seam[20, 16] | AGAGGCTTACGACGATAAAAACCATTTGCACCCATCCAGGAT |
| seam[21, 13] | CAGCTACACAAGATTAGTTGCTATAAATAGCGTCTGAACCGC |
| seam[22, 16] | ACTGGCTCGAATTACCTTATGCGAACAAAGTCCATCCAGGAT |
| seam[23, 13] | AGAGGGTATAACTGAACACCCTGATTTTAAGATCTGAACCGC |
| seam[24, 16] | GACAGATGCCGAACTGACCAACTTTTACGCAGCATCCAGGAT |
| seam[25, 13] | TATGTTAGTGATTAAGACTCCTTATGAAAGAGTCTGAACCGC |
| seam[26, 16] | CACTACGATTAAACGGGTAAAATATTGAGCCACATCCAGGAT |
| seam[27, 13] | TTTGGGAATATCACCGTCACCGACCGTAATGCTCTGAACCGC |
| seam[28, 16] | TGATACCGTCGAGGTGAATTTCTTCAGAGCCACATCCAGGAT |
| seam[29, 13] | CCACCGGATCAAAATCACCGGAACAAACAGCTTCTGAACCGC |
| seam[30, 16] | AAAGTTTTCCCTCATAGTTAGCGTGCGTCATACATCCAGGAT |
| seam[31, 13] | CATGGCTTTTTACCGTTCCAGTAAAACGATCTTCTGAACCGC |
| [1,2] | AACAGGAGGGAACCTATTATTCTGGCCCC CTG |
| [1,5] | TGCTTTCCAGAGGCTGAGACTCCTCTTGA GTA |
| [1,10] | GCTAGGGCATTAGCGGGGTTTTGCGTACT GGT |
| [1,14] | GAAAGGAAAAGTGCCGTCGAGAGGGTTGA TAT |
| [1,18] | AGCTTGACCCCGGAATAGGTGTATAGCATT CC |
| [1,22] | AATCGGAATTTAGTACCGCCACCCGTTTCG TC |
| [1,26] | GTTTTTTGCAGAACCGCCACCCTCGCCCA ATA |
| [32,4] | AAGTATTATCGTTAGAATCAGAGCTTAGAC AG |
| [32,8] | GGATTAGGGCTGGCAAACGAGCACAGTGT TTT |
| [32,12] | AGGCGGATGGGAAGAAAGCGAAAGAAAGA GTC |
| [32,16] | AAGTATAGGGGGAAAGCCGGCGAACGTG GCGA |
| [32,20] | TCAGGAGGCCCTAAAGGGAGCCCCTTGGA ACA |
| [32,24] | GCCACCCTGGGTCGAGGTGCCGTAACTCC AAC |
| [32,28] | CACCCTCATACGTGAACCATCACCCAGGG CGA |

**Table S3** 0.1X Origami

Sequences with red color are used to bind Cy5 fluorophores.

| 0[108] | AGTCACAATTCTACTAATAGCCTGGGGTTCCACC ATC CAG GAT |
| --- | --- |
| 0[118] | CTGACTATCATCAAAGTACCGAGCTCGATCA |
| 0[129] | TCAGGTTAACATCCAATAAATAAAGCATAC ATC CAG GAT |
| 0[13] | CCAACAGGCCAAAACAGGAAGATTGTATAAAGAC |
| 0[139] | TAAATCAAGCCTTCCTGACTCTAGAGGATTACGAGCCGGTT |
| 0[150] | ACGAGGGCAAGGCAAAGAATACTTTTGTAAAAAC ATC CAG GAT |
| 0[160] | CAGTTCAGCAACATTAGCTTGCATGCCTCAA |
| 0[171] | CAAATTTAAGCAATAAAGCCAACATTTCATATC ATC CAG GAT |
| 0[181] | TGAATCCCACAACCCGAAAACGACGGCCAGAA |
| 0[192] | TCTATATAAAGCTAACCCAGCAGGCGAGTCAAAGGC ATC CAG GAT |
| 0[202] | GCGAAAAAGAACAAATTTCCCAGTCACGAAT |
| 0[213] | CCAACAAATCCTGTTTGATGCAGCAAGGGTAAAC ATC CAG GAT |
| 0[223] | GAACGTGGAATGGGAAAGTTGGGTAACGTGT |
| 0[234] | GTCCAGAAATCGGCAAAATCCCTGGCTGAGAAC ATC CAG GAT |
| 0[24] | CGGATTTAGTTTGACCATCGTGCCAATCAGGC ATC CAG GAT |
| 0[244] | TTGGAACATAGATGGGGATGTGCTGCAAGAAA |
| 0[255] | TGTTAATCAAAAGAATAGCCAGCTGAGTCAAAC ATC CAG GAT |
| 0[265] | GATAGGGTCATCTGCACGCCAGCTGGCGGAG |
| 0[286] | AGCTTAATACGACAGTTGCGGGCCTCTTCTCA |
| 0[297] | CGGAACTAAAGTACGGTGTCGCCAGGGCTGATC ATC CAG GAT |
| 0[3] | GATTTCCCAATTCTGCGAAATCGGCCGAGAGAC ATC CAG GAT |
| 0[307] | AGGTCATTATCGCACCTGTTGGGAAGGGGTT |
| 0[318] | CCTTTTCATTCCATATAACAGGCGGGAGAGGC ATC CAG GAT |
| 0[328] | CCTTTAATGGCACCGCCGCCATTCAGGCTATG |
| 0[34] | AGCGAACCGCAAATACCGGTTGATAATCAAAG |
| 0[45] | TTCGTTTCGCAAATGGTCATTCCAGAGTCTGGC ATC CAG GAT |
| 0[55] | TCGCGTTTTAATATTCATGTCAATCATACCT |
| 0[66] | AAGACGTTTAGCTATATTTTCGTTGCGAATCGAC ATC CAG GAT |
| 0[76] | AGGAAGCCTTAAATTAACGGTAATCGTACA |
| 0[87] | CAAAAGCGCGAGCTGAAAAGAGCTAAATTGTTC ATC CAG GAT |
| 0[97] | GCGGATTGCATTTTTTATCATGGTCA |
| 1[276] | AACATGTTTTAATCACCATGATAT |
| 2[129] | AAGTGTAAAGTAGTAGCATCTTTACC |
| 3[109] | CAATGCCTAATGAGTGGTGGCATGAAGCAAA |
| 3[119] | ACAACACCCCGGAATAATTCGCGTCTGAAAT CTG AAC CGC |
| 3[14] | TCTACAGAAAAGCCCCAAGGCAAAGATT |
| 3[140] | TCAACGGCAGGTCGTAGCCAGCTTTCATAAAT CTG AAC CGC |
| 3[151] | GGACGGGAGAAGCCTTTCATACAAATGACCA |
| 3[161] | TTTTTAGTGCCAAAATGTGAGCGAGTACCTT CTG AAC CGC |
| 3[172] | CCCATGACCCTGTAATTAGCAAAAGCTTTAAA |
| 3[182] | ATTTTAACGTTGTTCGGATTCTCCGTGGCCGT CTG AAC CGC |
| 3[193] | GCACGGTTGTACCAAATCAGAGCCAATTCAT |
| 3[203] | GAGTAACCAGGGTCGGCGGATTGACCGTACTT CTG AAC CGC |
| 3[214] | GTACGGTCCACGCTGGTTTGCATATGCCT |
| 3[224] | GATTCAGCGATTTAGGTCACGTTGGTGAGAT CTG AAC CGC |
| 3[235] | GGGCCTGAGAGAGTTGGTGGTTCCCTATTAAA |
| 3[24] | GCTGCTGCATTAATGACGAGTAGAAGCAAACTT CTG AAC CGC |
| 3[245] | AGGCCGAAAGGGGCGCATCGTAACCGTGTGAGT CTG AAC CGC |
| 3[255] | ACATTGCCCTTCACCGCCTTATAGTTCCAGT |
| 3[266] | TCACCAGCTATTCAGTTTGAGGGGACGTGCTGAATATAATGGA |
| 3[276] | ATAGTGAGACGGGCAACCCTGTAGCTC |
| 3[287] | TCAACCCGATCGGATCGGCCTCAGGAAGTTTGT CTG AAC CGC |
| 3[297] | CTAGTGGTTTTTCTTTATATGCATGGCTTAG |
| 3[3] | TTTAACGCGCGGGGAGAGTTGATAGAGAGTAT CTG AAC CGC |
| 3[308] | AAATTAGCGCAATCCAGCCAGCTTTCCTGCTT CTG AAC CGC |
| 3[318] | CCGTTTGCGTATTGGGCTGGAAGTTTGATAAG |
| 3[329] | GTAGCTCGCCATTTTCTGGTGCCGGAAATCAGT CTG AAC CGC |
| 3[35] | TCATTGTGTACCTTTAAATTGTAAACGTTAA |
| 3[45] | GAGTCGGGAAACCTGTTAGATACAAGCTTCAAT CTG AAC CGC |
| 3[56] | AGCAAAAAACTAGTTGTTAAAATTCGCACGA |
| 3[66] | AGAGCTCACTGCCCGCTATAACCTTTCAAATAT CTG AAC CGC |
| 3[77] | TTCCTGTGTGAACTCACATTAATTGCATTTGGGAGATTAAG |
| 3[98] | ATCCGCATTCGTAAACCAATAGGAACGCTATT CTG AAC CGC |
| 4[86] | TAGCTGTTGTTTGTTAAATCAGCTCAT |

**Table S4** Tube 2 for 140 nm tube origami

| [01,06] | GAGAATAGGTCACCAGTACAAACTCCGCCACC |
| --- | --- |
| [01,10] | TGCTAAACTCCACAGACAGCCCTCTACCGCCA |
| [01,18] | TAAGCGTCGGTAATAAGTTTTAACCCGTCGAG |
| [01,22] | GGAAAGCGGTAACAGTGCCCGTATCGGGGTTT |
| [01,26] | ACAAACAACTGCCTATTTCGGAACCTGAGACT |
| [03,06] | AAAGGCCGCTCCAAAAGGAGCCTTAGCGGAGT |
| [03,10] | ATATATTCTCAGCTTGCTTTCGAGTGGGATTT |
| [03,18] | AACCAGAGACCCTCAGAACCGCCACGTTCCAG |
| [03,22] | GTTTGCCACCTCAGAGCCGCCACCGCCAGAAT |
| [03,26] | TCGGCATTCCGCCGCCAGCATTGATGATATTC |
| [05,06] | GCGAAACAAGAGGCTTTGAGGACTAGGGAGTT |
| [05,10] | CTCATCTTGGAAGTTTCCATTAAACATAACCG |
| [05,18] | GACTTGAGGTAGCACCATTACCATATCACCGG |
| [05,22] | TTATTCATGTCACCAATGAAACCATTATTAGC |
| [05,26] | ATTGAGGGAATCAGTAGCGACAGACGTTTTCA |
| [07,06] | CCAAATCATTACTTAGCCGGAACGTACCAAGC |
| [07,10] | AGTAATCTTCATAAGGGAACCGAACTAAAACA |
| [07,18] | TTATTACGTAAAGGTGGCAACATACCGTCACC |
| [07,22] | ATACCCAAACACCACGGAATAAGTGACGGAAA |
| [07,26] | GAAGGAAAAATAGAAAATTCATATTTCAACCG |
| [09,06] | AAAGATTCTAAATTGGGCTTGAGATTCATTAC |
| [09,10] | ACGAACTATTAATCATTGTGAATTTCATCAAG |
| [09,18] | TGAACAAAGATAACCCACAAGAATAAGACTCC |
| [09,22] | GCGCATTAATAAGAGCAAGAAACAATAACGGA |
| [09,26] | CTTTACAGTATCTTACCGAAGCCCAGTTACCA |
| [11,06] | TAAATATTGAGGCATAGTAAGAGCACAGGTAG |
| [11,10] | ACTGGATATCGTTTACCAGACGACTTAATAAA |
| [11,18] | TATTTTGCACGCTAACGAGCGTCTGAACACCC |
| [11,22] | AGGTTTTGGCCAGTTACAAAATAAACAGGGAA |
| [11,26] | GAGGCGTTTCCCAATCCAAATAAGATAGCAGC |
| [13,06] | TACCTTTAAGGTCTTTACCCTGACAATCGTCA |
| [13,10] | GAAGCAAAAAAGCGGATTGCATCAATGTTTAG |
| [13,18] | ATCGGCTGACCAAGTACCGCACTCTTAGTTGC |
| [13,22] | CTAATTTACCGTTTTTATTTTCATCTTGCGGG |
| [13,26] | TAAGTCCTGCGCCCAATAGCAAGCAAGAACGC |
| [15,06] | TTTCATTTCTGTAGCTCAACATGTTTAGAGAG |
| [15,10] | TCGCAAATAAGTACGGTGTCTGGACCAGACCG |
| [15,18] | CATATTTATTTCGAGCCAGTAATAAATCAATA |
| [15,22] | ACGCTCAACGACAAAAGGTAAAGTATCCCATC |
| [15,26] | GCGTTATACGACAATAAACAACATACAATAGA |
| [17,06] | TATATTTTCATACAGGCAAGGCAAAGCTATAT |
| [17,10] | CAACGCAAAGCAATAAAGCCTCAGGATACATT |
| [17,18] | ACAAAGAAAATTTCATCTTCTGACAGAATCGC |
| [17,22] | TATGTAAAGAAATACCGACCGTGTTAAAGCCA |
| [17,26] | TAACCTCCAATAAGAATAAACACCTATCATAT |
| [19,06] | CATGTCAAAAATCACCATCAATATAACCCTCA |
| [19,10] | AGAGAATCAGCTGATAAATTAATGCTTTATTT |
| [19,18] | AAATCAATCGTCGCTATTAATTAAATCGCAAG |
| [19,22] | TTGAATTATTGAAAACATAGCGATTATAACTA |
| [19,26] | AAAACAAACTGAGAAGAGTCAATATACCTTTT |
| [21,06] | ACCCGTCGTTAAATTGTAAACGTTAAAACTAG |
| [21,10] | CTTTCATCTCGCATTAAATTTTTGAGCAAACA |
| [21,18] | TTTAACGTTCGGGAGAAACAATAACAGTACAT |
| [21,22] | ACAGAAATCTTTGAATACCAAGTTAATTTCAT |
| [21,26] | AACCTACCGCGAATTATTCATTTCACATCAAG |
| [23,06] | GGCGATCGCGCATCGTAACCGTGCGAGTAACA |
| [23,10] | TTCGCCATGGACGACGACAGTATCGTAGCCAG |
| [23,18] | TTATTAATGAACAAAGAAACCACCTTTTCAGG |
| [23,22] | CGACAACTTCATCATATTCCTGATCACGTAAA |
| [23,26] | GGATTTAGTTCATCAATATAATCCAGGGTTAG |
| [25,06] | GCTCACAAGGGTAACGCCAGGGTTTTGGGAAG |
| [25,10] | TCATAGCTTGTAAAACGACGGCCAAAGCGCCA |
| [25,18] | CTAAAGCAAATCAATATCTGGTCACCCGAACG |
| [25,22] | GCCACGCTTTGAAAGGAATTGAGGAAACAATT |
| [25,26] | AGGCGGTCTCTTTAGGAGCACTAAACATTTGA |
| [27,06] | AGCTGATTACTCACATTAATTGCGTGTTATCC |
| [27,10] | TGGTTTTTCTTTCCAGTCGGGAAAAATCATGG |
| [27,18] | GCCAACAGATACGTGGCACAGACATGAAAAAT |
| [27,22] | GTCACACGATTAGTCTTTAATGCGGCAACAGT |
| [27,26] | GAAATGGAAAACATCGCCATTAAACAGAGGTG |
| [29,06] | TATCAGGGCGAAAATCCTGTTTGACGGGCAAC |
| [29,10] | TGGACTCCGGCAAAATCCCTTATACGCCAGGG |
| [29,18] | GTTGTAGCCCTGAGTAGAAGAACTACATTCTG |
| [29,22] | GTAAAAGACTGGTAATATCCAGAAATTCACCA |
| [29,26] | AGAAGTGTCATTGCAACAGGAAAAAATCGTCT |
| [16,16] | AGAGGCATACAACGCCAACATGTATCTGCGAA |
| [18,13] | CTGTAATAGGTTGTACCAAAAACACAAATATA |
| [18,16] | TTTTAGTTCGCGAGAAAACTTTTTTTATGACC |
| [20,13] | TCAGGTCATTTTTGAGAGATCTACCCTTGCTT |
| [20,16] | CTGTAAATATATGTGAGTGAATAAAAAGGCTA |
| [22,13] | AAATAATTTTTAACCAATAGGAACAACAGTAC |
| [22,16] | CTTTTACACAGATGAATATACAGTGCCATCAA |
| [24,13] | GCTTCTGGCACTCCAGCCAGCTTTACATTATC |
| [24,16] | ATTTTGCGTTTAAAAGTTTGAGTACCGGCACC |
| [26,13] | CCCGGGTACCTGCAGGTCGACTCTCAAATATC |
| [26,16] | AAACCCTCTCACCTTGCTGAACCTAGAGGATC |
| [28,13] | GGGAGAGGCATTAATGAATCGGCCACCTGAAA |
| [28,16] | GCGTAAGAAGATAGAACCCTTCTGAACGCGCG |
| [30,13] | AGTTTGGACGAGATAGGGTTGAGTGTAATAAC |
| [30,16] | ATCACTTGAATACTTCTTTGATTAGTTGTTCC |
| [02,04] | TGAGTTTCAAAGGAACAACTAAAGATCTCCAA |
| [02,08] | TGTAGCATAACTTTCAACAGTTTCTAATTGTA |
| [02,12] | CGTAACGAAAATGAATTTTCTGTAGTGAATTT |
| [02,20] | TGCCTTGACAGTCTCTGAATTTACCCCTCAGA |
| [02,24] | AATGCCCCATAAATCCTCATTAAAAGAACCAC |
| [04,04] | AAAAAAGGCTTTTGCGGGATCGTCGGGTAGCA |
| [04,08] | TCGGTTTAGGTCGCTGAGGCTTGCAAAGACTT |
| [04,12] | CTTAAACAACAACCATCGCCCACGCGGGTAAA |
| [04,20] | GCCACCACTCTTTTCATAATCAAATAGCAAGG |
| [04,24] | CACCAGAGTTCGGTCATAGCCCCCTCGATAGC |
| [06,04] | ACGGCTACAAGTACAACGGAGATTCGCGACCT |
| [06,08] | TTTCATGATGACCCCCAGCGATTAAGGCGCAG |
| [06,12] | ATACGTAAGAGGCAAAAGAATACACTGACCAA |
| [06,20] | CCGGAAACTAAAGGTGAATTATCATAAAAGAA |
| [06,24] | AGCACCGTAGGGAAGGTAAATATTTTATTTTG |
| [08,04] | GCTCCATGACGTAACAAAGCTGCTACACCAGA |
| [08,08] | ACGGTCAATGACAAGAACCGGATATGGTTTAA |
| [08,12] | CTTTGAAAATAGGCTGGCTGACCTACCTTATG |
| [08,20] | ACGCAAAGAAGAACTGGCATGATTTGAGTTAA |
| [08,24] | TCACAATCCCGAGGAAACGCAATAATGAAATA |
| [10,04] | ACGAGTAGATCAGTTGAGATTTAGCGCCAAAA |
| [10,08] | TTTCAACTACGGAACAACATTATTAACACTAT |
| [10,12] | CGATTTTAGGAAGAAAAATCTACGGATAAAAA |
| [10,20] | GCCCAATAGACGGGAGAATTAACTTTCCAGAG |
| [10,24] | GCAATAGCAGAGAATAACATAAAAACAGCCAT |
| [12,04] | GGAATTACCATTGAATCCCCCTCACCATAAAT |
| [12,08] | CATAACCCGCGTCCAATACTGCGGTATTATAG |
| [12,12] | CCAAAATAAGGGGGTAATAGTAAAAAAAGATT |
| [12,20] | CCTAATTTAAGCCTTAAATCAAGAATCGAGAA |
| [12,24] | ATTATTTATTAGCGAACCTCCCGACGTAGGAA |
| [14,04] | CAAAAATCATTGCTCCTTTTGATAATTGCTGA |
| [14,08] | TCAGAAGCCTCCAACAGGTCAGGATTTAAATA |
| [14,12] | AAGAGGAACGAGCTTCAAAGCGAAAGTTTCAT |
| [14,20] | CAAGCAAGCGAGCATGTAGAAACCAGAGAATA |
| [14,24] | TCATTACCGAACAAGAAAAATAATAATTCTGT |
| [16,04] | ATATAATGGGGGCGCGAGCTGAAATTAACATC |
| [16,08] | TGCAACTAGGTCAATAACCTGTTTAGAATTAG |
| [16,12] | TCCATATATTTAGTTTGACCATTAAGCATAAA |
| [16,20] | TAAAGTACCAGTAGGGCTTAATTGCTAAATTT |
| [16,24] | CCAGACGACAAATTCTTACCAGTAGATAAATA |
| [18,04] | CAATAAATAAATGCAATGCCTGAGAAGGCCGG |
| [18,08] | CAAAATTAGGATAAAAATTTTTAGGATATTCA |
| [18,12] | GCTAAATCCTTTTGCGGGAGAAGCCCGGAGAG |
| [18,20] | AATGGTTTTGCTGATGCAAATCCATTTTCCCT |
| [18,24] | AGGCGTTAGGCTTAGGTTGGGTTAAGCTTAGA |
| [20,04] | AGACAGTCTCATATGTACCCCGGTTTGTATAA |
| [20,08] | ACCGTTCTGATGAACGGTAATCGTAATATTTT |
| [20,12] | GGTAGCTATTGCCTGAGAGTCTGGTTAAATCA |
| [20,20] | TAGAATCCCCTTTTTTAATGGAAACGGATTCG |
| [20,24] | TTAAGACGATTAATTACATTTAACACAAAATC |
| [22,04] | GCAAATATGATTCTCCGTGGGAACCGTTGGTG |
| [22,08] | GTTAAAATAACATTAAATGTGAGCATCTGCCA |
| [22,12] | GCTCATTTCGCGTCTGGCCTTCCTGGCCTCAG |
| [22,20] | CCTGATTGAAAGAAATTGCGTAGAAGAAGGAG |
| [22,24] | GCGCAGAGATATCAAAATTATTTGTATCAGAT |
| [24,04] | TAGATGGGGTGCGGGCCTCTTCGCGCAAGGCG |
| [24,08] | GTTTGAGGTCAGGCTGCGCAACTGTTCCCAGT |
| [24,12] | GAAGATCGTGCCGGAAACCAGGCAGTGCCAAG |
| [24,20] | CGGAATTACGTATTAAATCCTTTGGTTGGCAA |
| [24,24] | GATGGCAAAAGTATTAGACTTTACAAGGTTAT |
| [26,04] | ATTAAGTTTTCCACACAACATACGCCTAATGA |
| [26,08] | CACGACGTGTTTCCTGTGTGAAATTTGCGCTC |
| [26,12] | CTTGCATGCCGAGCTCGAATTCGTCCTGTCGT |
| [26,20] | ATCAACAGGAGAGCCAGCAGCAAAATATTTTT |
| [26,24] | CTAAAATAAGTATTAACACCGCCTCGAACTGA |
| [28,04] | GTGAGCTAGCCCTTCACCGCCTGGGGTTTGCC |
| [28,08] | ACTGCCCGCTTTTCACCAGTGAGATGGTGGTT |
| [28,12] | GCCAGCTGCGGTTTGCGTATTGGGAATCAAAA |
| [28,20] | GAATGGCTACCAGTAATAAAAGGGCAAACTAT |
| [28,24] | TAGCCCTATTATTTACATTGGCAGCAATATTA |
| [30,04] | CCAGCAGGCGATGGCCCACTACGTGAGGTGCC |
| [30,08] | CCGAAATCAACGTCAAAGGGCGAAAAGGGAGC |
| [30,12] | GAATAGCCACAAGAGTCCACTATTAAGCCGGC |
| [30,20] | CGGCCTTGGTCTGTCCATCACGCATTGACGAG |
| [30,24] | CCGCCAGCTTTTATAATCAGTGAGAGAATCAG |
| [02,13] | ACGTTAGTTCTAAAGTTTTGTCGTGATACAGG |
| [02,16] | AGTGTACTATACATGGCTTTTGATCTTTCCAG |
| [04,13] | CAATGACAGCTTGATACCGATAGTCTCCCTCA |
| [04,16] | GAGCCGCCCCACCACCGGAACCGCTGCGCCGA |
| [06,13] | AAACGAAATGCCACTACGAAGGCAGCCAGCAA |
| [06,16] | AATCACCACCATTTGGGAATTAGACCAACCTA |
| [08,13] | CCAGGCGCGAGGACAGATGAACGGGTAGAAAA |
| [08,16] | TACATACACAGTATGTTAGCAAACTGTACAGA |
| [10,13] | GGACGTTGAGAACTGGCTCATTATGCGCTAAT |
| [10,16] | ATCAGAGAGTCAGAGGGTAATTGAACCAGTCA |
| [12,13] | TTTGCCAGGCGAGAGGCTTTTGCAATCCTGAA |
| [12,16] | TCTTACCAACCCAGCTACAATTTTAAAGAAGT |
| [14,13] | TTTTAATTGCCCGAAAGACTTCAACAAGAACG |
| [14,16] | GGTATTAATCTTTCCTTATCATTCATATCGCG |
| [16,13] | CGAGTAGAACAGTTGATTCCCAATATTTAGGC |
| hlinker1 | CAGCGAAAGCGTCAGACTGTAGCGATCAAGTT |
| hlinker2 | GAATAAGGGCAGATAGCCGAACAATTTTTAAG |
| hlinker3 | AAACAGTTGCTTATCCGGTATTCTAAATCAGA |
| hlinker4 | TCAATTCTGAAAAAGCCTGTTTAGGGAATCAT |
| hlinker5 | AGAAAAGCGAAGATGATGAAACAAAATTACCT |
| hlinker6 | CAGCTGGCGCCGTCAATAGATAATCAACTAAT |
| hlinker7 | GAGTTGCACTACATTTTGACGCTCACGCTCAT |
| hlinker8 | TGCCTTTAGACAGCATCGGAACGAACCCTCAG |
| hlinker9 | AAAAGTAACTTGCCCTGACGAGAACATTCAGT |
| hlinker10 | TATAGAAGCAGAAAACGAGAATGAAATGCTTT |
| hlinker11 | AATTACTAACTAATAGTAGTAGCAAGGTGGCA |
| hlinker12 | GAGCAAAACCCAAAAACAGGAAGATGATAATC |
| hlinker13 | AGATTAGAGAAAGGGGGATGTGCTTATTACG |

**Table S5** Linkers between tube 1 and 2

| [06, 28] | CACACAACGACAGCATCGGAACGAACCCTCAG |
| --- | --- |
| [08, 28] | CGGGCCTCAAATTGTGTCGAAATCTGTATCAT |
| [10, 28] | TCTCCGTGCTTGCCCTGACGAGAACATTCAGT |
| [12, 28] | TATGTACCTAATGCAGATACATAAGAATACCA |
| [14, 28] | TGCAATGCCAGAAAACGAGAATGAAATGCTTT |
| [16, 28] | GCGCGAGCGATGGCTTAGAGCTTAAGAGGTCA |
| [18, 28] | GCTCCTTTACTAATAGTAGTAGCAAGGTGGCA |
| [20, 28] | TGAATCCCATTCAAAAGGGTGAGATAATGTGT |
| [22, 28] | AGTTGAGACCCAAAAACAGGAAGATGATAATC |
| [24, 28] | TAACAAAGGTAATGGGATAGGTCAAAACGGCG |
| [26, 28] | TACAACGGGAAAGGGGGATGTGCTTATTACGC |
| [28, 28] | TTGCGGGATGTAAAGCCTGGGGTGAGCCGGAA |
| [30, 28] | GGAACAACGCAAGCGGTCCACGCTCCCTGAGA |
| [03, 01] | AATAATAACCTGGCCCTGAGAGAGTGATTGCC |
| [05, 01] | CAGCGAAAATACGAGCCGGAAGCACACAATTC |
| [07, 01] | CGCCTGATTTCGCTATTACGCCAGGATCGGTG |
| [09, 01] | GAATAAGGGGAACAAACGGCGGATCGTCGGAT |
| [11, 01] | CATTCAACCCGGTTGATAATCAGAGTCAATCA |
| [13, 01] | AAACAGTTCTGAGTAATGTGTAGGATTTTAAA |
| [15, 01] | TTTTTGCGTGAAAAGGTGGCATCACATTTGGG |
| [17, 01] | TCAATTCTTGATAAGAGGTCATTTCTTTAATT |
| [19, 01] | AGGTAAAGCCTCAAATGCTTTAAAATATTCAT |
| [21, 01] | AGAAAAGCTTTAGGAATACCACATGATTCATC |
| [23, 01] | GATTGACCCTGCTCATTCAGTGAAAATCAACG |
| [25, 01] | CAGCTGGCAGATTTGTATCATCGCAAACAAAG |
| [27, 01] | GCATAAAGTCGTCACCCTCAGCAGGGCCGCTT |

**REFERENCES**

[1] H. Qiu, F. Li, Y. Du, R. Li, J.Y. Hyun, S.Y. Lee, J.H. Choi, Programmable Aggregation of Artificial Cells with DNA Signals, ACS Synth Biol, 10 (2021) 1268-1276.

[2] F. Chen, L. Zhu, Y. Zhang, D. Kumar, G. Cao, X. Hu, Z. Liang, S. Kuang, R. Xue, C. Gong, Clathrin-mediated endocytosis is a candidate entry sorting mechanism for Bombyx mori cypovirus, Scientific Reports, 8 (2018).
